# Host systemic metabolism and cancer metabolic vulnerabilities: mechanisms and therapeutic opportunities

**DOI:** 10.3389/fonc.2026.1897564

**Published:** 2026-07-28

**Authors:** Rashid Mir, Jameel Barnawi, Naseh A Algehainy, Mohammed M. Jalal, Malik A. Altayar, Reema M Almotairi, Faris J Tayeb, Mamdoh S Moawadh, Ruqaiah I Bedaiwi, Kholoud S. Almasoudi, Ibrahim Altedlawi Albalawi, Abdulrahman Alasmari, Mohammad Muzaffar Mir

**Affiliations:** 1 Prince Fahad Bin Sultan Chair for Biomedical Research, University of Tabuk, Tabuk, Saudi Arabia; 2 Department of Medical Laboratory Technology, Faculty of Applied Medical Sciences, University of Tabuk, Tabuk, Saudi Arabia; 3 Department of Surgical Oncology, Faculty of Medicine, University of Tabuk, Tabuk, Saudi Arabia; 4 Department of Clinical Biochemistry, College of Medicine, University of Bisha, Bisha, Tabuk, Saudi Arabia

**Keywords:** cancer therapeutics, epigenetic regulation, HIF-1α, metabolic reprogramming, mitochondrial dynamics, oncometabolites, tumour microenvironment, warburg effect

## Abstract

**Background:**

Central molecular mediators—including hypoxia-inducible factors (HIF-1α/HIF-2α), MYC, wild-type and mutant p53, NF-κB, STAT3, SREBPs, NRF2, and KRAS—orchestrate these pathways by linking nutrient availability to oncogenic signalling, epigenetic reprogramming, and immune-metabolic crosstalk within the tumour microenvironment. Key metabolic enzymes including HK2, PKM2, LDH-A, IDH1/2, GLS1, and FASN serve as direct effectors and therapeutic targets. Mitochondrial dynamics—biogenesis (PGC-1α), fission (DRP1), fusion (MFN1/2, OPA1), and mitophagy (PINK1-Parkin)—constitute a critical regulatory layer. The bidirectional epigenetic-metabolic axis, mediated by acetyl-CoA, SAM, α-ketoglutarate, 2-hydroxyglutarate, and lysine lactylation, amplifies oncogenic transcriptional programs and locks cells into malignant states. Central to this review is the thesis that metabolic plasticity—the capacity of cancer cells to dynamically switch between and co-opt multiple metabolic programs—is the primary driver of tumour progression, immune evasion, and resistance to therapy. Understanding and targeting this plasticity represents the central translational challenge of cancer metabolic oncology.

**Methods:**

A comprehensive narrative literature review was conducted across PubMed, Scopus, and Web of Science (2015–2025) using terms including metabolic reprogramming, Warburg effect, oncometabolites, mitochondrial dynamics, epigenetic metabolism, immunometabolism, and metabolic therapeutics. Peer-reviewed primary research and comprehensive reviews were evaluated. Limitations include restriction to English-language literature (2015–2025), potential publication bias toward high-impact journals, and the rapidly evolving nature of the field.

**Conclusion:**

Metabolic reprogramming is governed by an interconnected network of transcription factors, signalling cascades, epigenetic regulators, mitochondrial dynamics, and TME-immune crosstalk. FDA-validated targets include IDH1/2 (ivosidenib, enasidenib, vorasidenib—August 2024), HIF-2α (belzutifan), and mTOR (everolimus). An expanding clinical pipeline encompasses GLS1, MCT1, OXPHOS Complex I, FASN, and metabolic immune checkpoints. Future advances require single-cell/spatial metabolomics, AI-driven patient stratification, and rational combination strategies that preempt adaptive metabolic escape. Future advances require AI-driven genome-scale metabolic modelling for patient stratification, single-cell and spatial metabolomics to resolve intra-tumoral metabolic heterogeneity, and rational combination strategies targeting multiple metabolic nodes simultaneously to preempt adaptive resistance. Integration of circadian pharmacology, host metabolic comorbidity management (obesity, diabetes, gut microbiome modulation), and TME metabolic normalisation into cancer treatment frameworks will drive the next generation of precision metabolic oncology.

## 1 Introduction

Cancer is increasingly recognised as a metabolic disease. Metabolic reprogramming, the global rewiring of nutrient acquisition, energy production, and biosynthetic pathways, enables tumour cells to sustain uncontrolled proliferation, resist apoptosis, adapt to fluctuating microenvironmental conditions, and evade immune destruction (1–5). The classical Warburg effect describes the preferential use of aerobic glycolysis by cancer cells despite the availability of sufficient oxygen (6). However, modern evidence points to a much more complex and heterogeneous metabolic landscape that includes glycolysis, OXPHOS, glutaminolysis, fatty acid metabolism, one-carbon metabolism and the pentose phosphate pathway (PPP), regulated by the interaction of genetic, epigenetic and microenvironmental factors (7, 8). In contrast to normal differentiated cells that tightly couple nutrient availability with energy production and biomass synthesis, cancer cells uncouple these processes, using nutrients well in excess of immediate energy requirements to fuel biosynthetic fluxes that support rapid proliferation (2). This metabolic uncoupling is controlled by oncogene-driven transcriptional rewiring that constitutively activates anabolic programs irrespective of the availability of extracellular nutrients, resulting in the characteristic metabolic phenotype of malignancy. Metabolic reprogramming is now recognized as an early event in carcinogenesis, occurring before the onset of overt histological transformation in premalignant lesions, and is thus an attractive target for cancer interception strategies (3).

**Figure 1:**
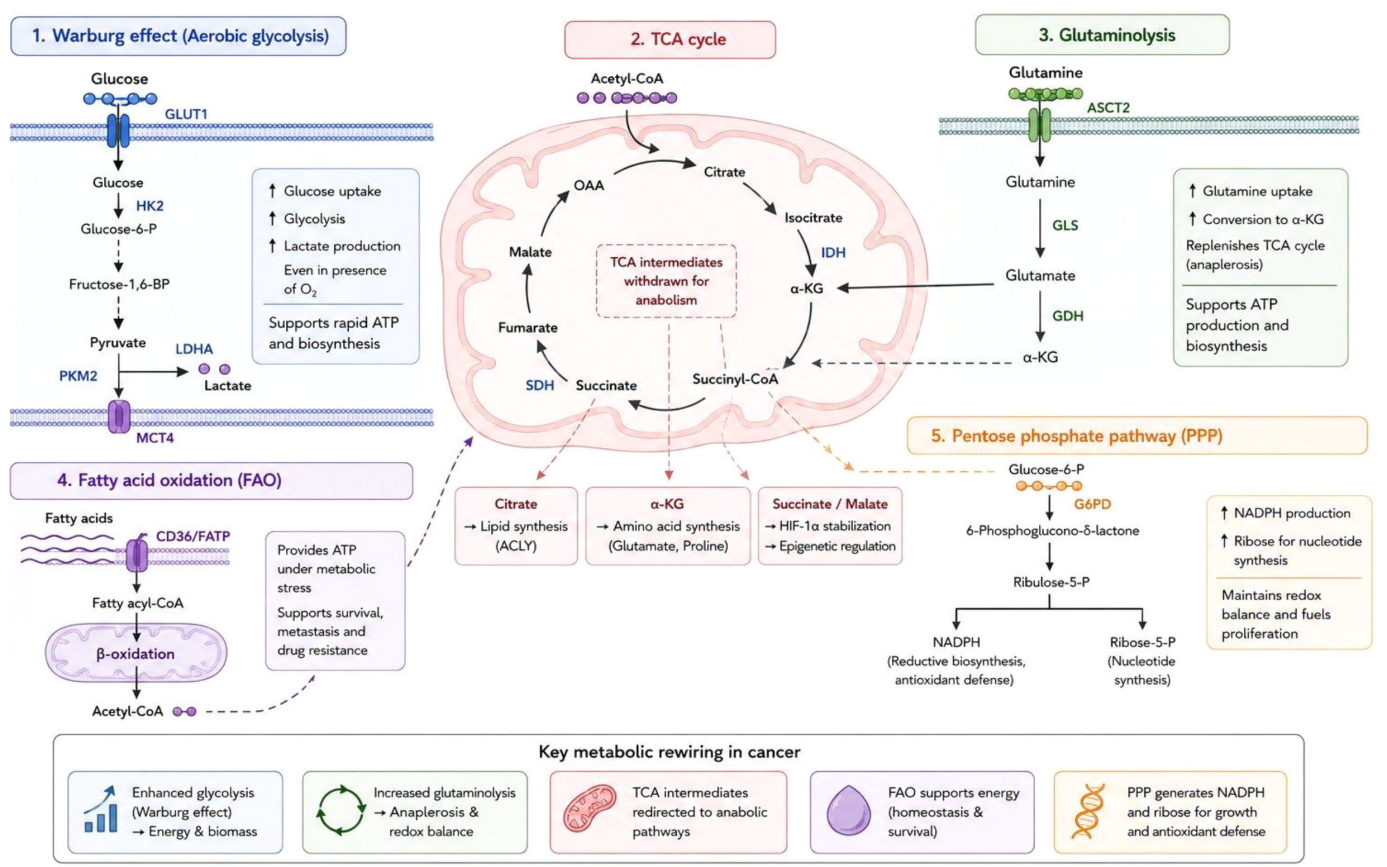
Central metabolic pathways rewired in cancer and key pharmacological inhibitors. Schematic illustrating the Warburg effect (aerobic glycolysis via GLUT1, HK2, PKM2, LDHA), TCA cycle with anabolic intermediate withdrawal, glutaminolysis (GLS, GDH), fatty acid oxidation (FAO via CD36, β-oxidation), and the pentose phosphate pathway (PPP via G6PD) generating NADPH and ribose for proliferation. Key biosynthetic outputs and regulatory enzymes are annotated. Specific drug/inhibitor mechanisms are annotated at their respective pathway nodes: (1) 2-Deoxyglucose (2-DG) and Lonidamine inhibit HK2, blocking the committed glycolytic step and disrupting anti-apoptotic HK2–VDAC1 interaction at the outer mitochondrial membrane; (2) TEPP-46 (ML265) activates PKM2 tetrameric assembly, redirecting glycolytic intermediates away from biosynthetic pathways and suppressing nuclear PKM2 coactivator activity; (3) FX11 and GNE-140 inhibit LDH-A, blocking NAD^+^ regeneration and lactate export via MCT4, thereby suppressing TME acidification and restoring tumour-infiltrating lymphocyte (TIL) metabolic function; (6) CB-839 (Telaglenastat) inhibits GLS1, disrupting glutaminolysis-driven TCA anaplerosis, with synergistic annotations for mTOR and glycolysis co-inhibition; (7) TVB-2640 inhibits FASN, blocking de novo fatty acid synthesis and ferroptosis protection via lipid droplet depletion; (8) AZD3965 inhibits MCT1, disrupting glycolytic-oxidative lactate symbiosis between glycolytic and OXPHOS-competent cells in the TME. All figures in this manuscript were generated using ChatGPT (OpenAI, 2024) as an AI-assisted illustration tool.

**Figure 2:**
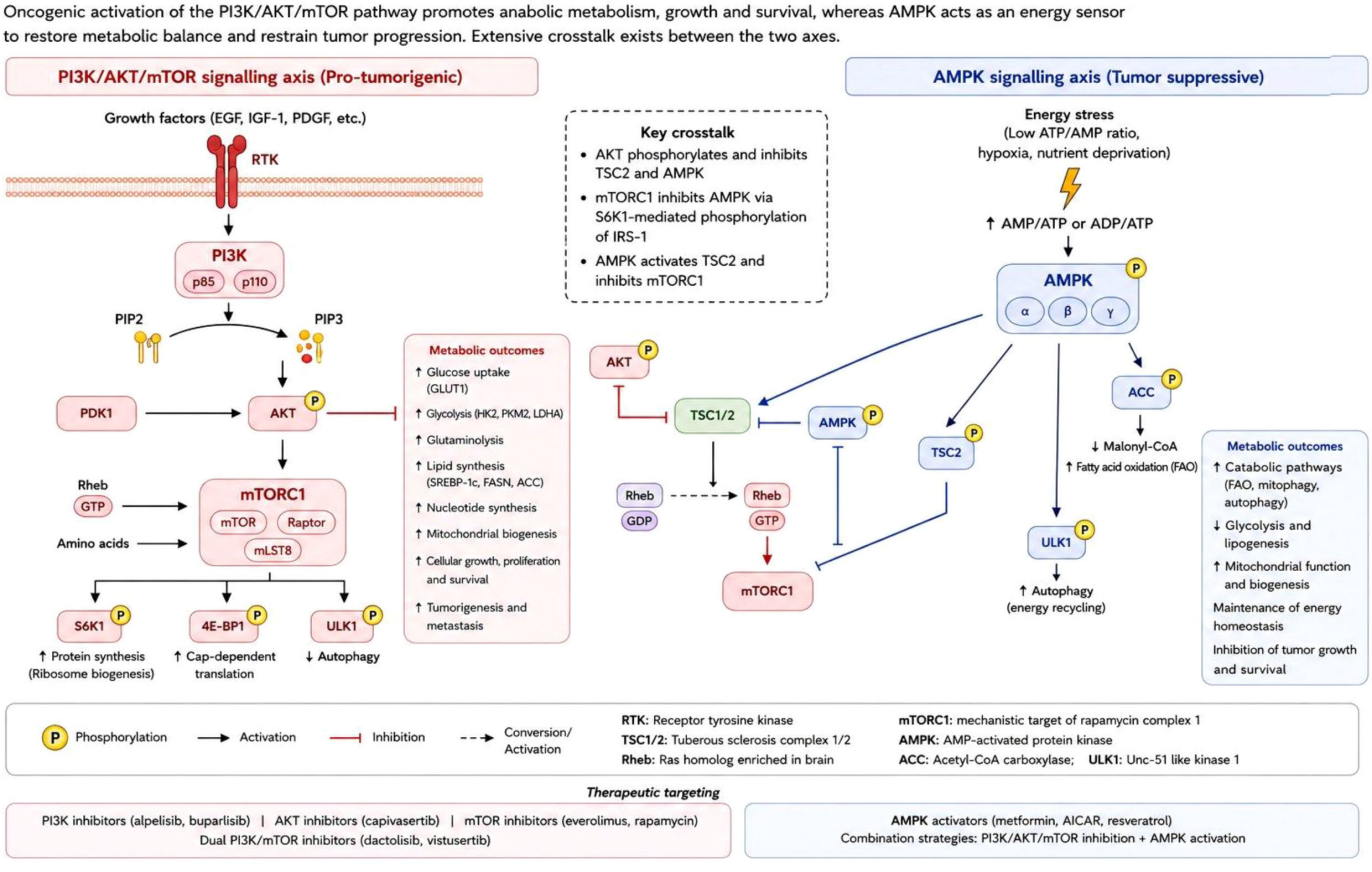
PI3K/AKT/mTOR and AMPK signalling axes in cancer metabolism. Left panel: oncogenic PI3K/AKT/mTOR cascade (RTK → PI3K → PIP3 → AKT → mTORC1) drives glucose uptake (GLUT1 upregulation), glycolysis (HK2, PFKFB3), lipid synthesis (SREBP1/2-driven FASN/ACC activation), nucleotide synthesis, and mitochondrial biogenesis (PGC-1α). Right panel: AMPK signalling axis (energy stress or metformin/AICAR → AMPK activation via LKB1/CaMKKβ) suppresses mTORC1, activates FAO (ACC phosphorylation), induces autophagy (ULK1 phosphorylation), and restores energy homeostasis. Inhibitory nodes annotated: alpelisib (PI3Kα), ipatasertib (AKT), everolimus/temsirolimus (mTORC1 rapalogs), metformin/AICAR (AMPK activators). Crosstalk between the two axes — wherein mTORC1 phosphorylates and inhibits IRS-1 as a negative feedback and AMPK phosphorylates TSC2 to suppress mTORC1 — is indicated by bidirectional arrows. Generated using ChatGPT (OpenAI, 2024).

Oncogenes including MYC, KRAS, and the PI3K/AKT/mTOR axis, and tumour suppressors p53, LKB1, and PTEN, directly rewire metabolic fluxes (9, 10). Extrinsic factors, hypoxia, acidosis, nutrient deprivation, and stromal crosstalk, further dictate metabolic dependencies in a spatially heterogeneous manner. Systemic host metabolism, including obesity, diabetes, and gut microbiome composition, also profoundly influences tumour energetics and therapy response (11, 12). Importantly, cancer metabolism extends to the tumour microenvironment (TME), where cancer- associated fibroblasts (CAFs), tumour-associated macrophages (TAMs), T cells, and dendritic cells (DCs) engage in metabolic symbiosis (reverse Warburg effect) and competition that shapes immune evasion and progression (13, 14) (**Figures 1**, **2**).

This review provides a comprehensive and critically integrated analysis of the molecular mediators of cancer metabolic reprogramming. We examine transcription factors (HIF-1α/2α, MYC, p53, NF-κB, STAT3, SREBPs, NRF2, KRAS), oncogenic signaling cascades (PI3K/AKT/mTOR (15–17), AMPK (18, 19), MAPK (20–22), Wnt (23, 24), Hippo (25, 26)), rate-limiting enzymes (HK2, PKM2, LDH-A, IDH1/2, GLS1, FASN), mitochondrial dynamics, epigenetic-metabolic crosstalk, and immunemetabolic interplay within the TME. We critically address controversies including the glycolysis– OXPHOS dichotomy, context-dependent roles of FAO, and wild-type versus mutant p53 metabolic switching. We further highlight emerging frontiers, single-cell and spatial metabolomics, ferroptosis, metabolic immune checkpoints, AI-driven discovery, and circadian-metabolism intersection, and their translational implications. Transcription factors and molecular regulators are summarised in **Table 1**; key signaling pathways in **Table 2** (**Figures 1**, **2**).

**Table 1:** Transcription factors and molecular regulators of cancer metabolic reprogramming.

Regulator	Metabolic pathways regulated	Key downstream effects	Therapeutic targets/agents	References
HIF-1α	Glycolysis↑ (GLUT1, HK2, LDHA, PKM2); PPP↑; FAO↓; PDK1↑	Warburg effect, lactate production, hypoxia adaptation	Acriflavine; 2-DG; AZD3965 (MCT1); HIF dimerization inhibitors	
HIF-2α	OXPHOS maintenance; iron homeostasis↑; lipid remodelling; MYC cooperation	Chronic hypoxia adaptation, angiogenesis, therapy resistance	Belzutifan/PT2977 (FDA-approved, 2021); PT2385	
MYC	Glycolysis↑ (GLUT1/3, HK2, LDHA); Glutaminolysis↑ (GLS1 via miR-23a/b); OXPHOS↑ (TFAM); Nucleotide synthesis↑	Glutamine addiction, anabolic growth, metabolic plasticity	BET inhibitors (JQ1, OTX015); GLS1 inhibitors (CB-839); indirect MYC targeting	
p53 (WT vs. mutant)	WT: OXPHOS↑ (SCO2, GLS2), glycolysis↓ (TIGAR). Mutp53: Glycolysis↑, lipogenesis↑ (SREBP1/2), PPP/NADPH↑	WT suppresses Warburg; mutp53 drives glycolysis, lipogenesis, therapy resistance	APR-246 (p53 reactivation); FASN inhibitors; statins (mutp53-mevalonate)	
NF-κB	Glycolysis↑ (GLUT1, HK2, PFK, LDHA); Glutamine/lipid metabolism↑	Aerobic glycolysis, lactate accumulation, immune evasion	IKK inhibitors; PI3K/AKT upstream targeting	
STAT3	Glycolysis↑ and OXPHOS↑ (dual); CSC maintenance via IL-6/STAT3 axis	Metabolic plasticity, immunosuppressive TME, stemness	Napabucasin; ruxolitinib (JAK-STAT); STAT3 inhibitors	
NRF2/KRAS/SREBPs	NRF2: PPP↑ (G6PD), GSH↑, NADPH↑. KRAS: glucose↑, macropinocytosis. SREBPs: lipogenesis↑ (FASN, ACC, SCD1, HMGCR)	ROS resistance, chemoresistance; nutrient scavenging; membrane biogenesis, ferroptosis protection	Brusatol (NRF2); sotorasib/adagrasib (KRAS G12C); fatostatin; TVB-2640 (FASN)	

References: 1–3, 6–14, 27–46.

**Table 2:** Key oncogenic signalling pathways driving cancer metabolic reprogramming.

Pathway	Key activators	Metabolic effects	Clinical agents (status)	Refs
PI3K/AKT/mTOR	RTK, PTEN loss, RAS	Glycolysis↑, SREBP-driven lipogenesis↑, autophagy↓, protein synthesis↑	Everolimus, temsirolimus (FDA-approved); alpelisib (PI3Kα); ipatasertib (AKT)	
AMPK	LKB1, CaMKKβ, energy stress	mTOR inhibition; FAO↑; glycolysis modulation; autophagy↑	Metformin (Phase II/III); AICAR; O304	
MAPK (ERK/JNK/p38)	RAS, growth factors, stress	Glycolysis↑ (PFKFB3); nucleotide synthesis↑ (CTPS1 via ERK2)	Trametinib + dabrafenib (FDA-approved); selumetinib (MEK1/2)	
Wnt/β-catenin	APC/AXIN loss, GSK3β inhibition	Glycolysis↑ (GLUT1, LDHA via WNT7B); FAO↑ (HCC)	LGK-974 (porcupine inhibitor, Phase I); β-catenin/TCF disruptors	
Hippo/YAP-TAZ	LATS1/2 inactivation, mechanotransduction	Glucose uptake↑; glycolytic enzyme induction; mitochondrial dynamics	Verteporfin (YAP/TEAD); TEAD inhibitors (Phase I)	

References: 15–26.

## 2 Central pathways in cancer metabolic reprogramming

Cancer cell metabolism is based on a highly interconnected network, not on single pathways, which leads to compensatory plasticity that underlies therapy resistance (27, 28). The core pathways, glycolysis, TCA cycle, OXPHOS, PPP, glutaminolysis, FAO, and *de novo* lipogenesis, function as an integrated system, and their interplay is summarised in **Figure 1**.

### 2.1 Glycolysis, warburg effect, and lactate biology

HIF-1α stabilised under hypoxia drives comprehensive glycolytic gene induction: GLUT1, GLUT3, HK2, PFK, PKM2, LDHA, MCT4, and PDK1 (suppressing pyruvate entry into TCA) (1, 29). Lactate is now recognised as a pleiotropic TME molecule: it stabilises HIF-1α normoxically via PHD inhibition, suppresses cytotoxic T and NK cell glycolysis, promotes M2-like macrophage polarisation via histone lactylation, and supports oxidative tumour cells via reverse Warburg lactate symbiosis (30). A key controversy is the glycolysis–OXPHOS dichotomy: while HIF-1α promotes glycolysis, MYC and HIF-2α sustain OXPHOS, and cancer stem cells (CSCs) predominantly rely on OXPHOS, remaining resistant to glycolysis-targeted therapies (31). Beyond the TME, circulating lactate contributes to systemic metabolic reprogramming, influencing liver gluconeogenesis, adipose lipolysis, and immune cell polarisation in distant tissues. This systemic dimension of lactate biology, sometimes termed the ‘lactate shuttle’, represents an emerging therapeutic dimension, as MCT inhibitors targeting intratumoural lactate dynamics may have offtarget systemic effects requiring careful dose modulation (31, 47). The clinical relevance of the glycolysis–OXPHOS dichotomy is most evident in haematological malignancies: AML blasts show high OXPHOS dependency targetable by IACS-010759, while glycolytic dominance characterises DLBCL subtypes responsive to 2-DG. Context-specific metabolic patient stratification is therefore essential for rational therapeutic trial design.

Critically, glycolysis and OXPHOS are not mutually exclusive programmes but dynamically co-exist within individual tumours: HIF-1α and HIF-2α operate as parallel transcriptional programmes activated under acute versus chronic hypoxia respectively, and MYC simultaneously drives glycolytic gene expression and mitochondrial biogenesis (via TFAM and PGC-1α), generating cells with elevated flux through both pathways. This metabolic coexistence underlies intra-tumoural metabolic heterogeneity and explains why monotherapy targeting either pathway alone is insufficient for durable tumour control. Cancer stem cells (CSCs) exploit OXPHOS dominance to evade glycolysis-targeted agents, creating drug-tolerant persister populations that serve as seeds of relapse — a clinically critical mechanism most clearly demonstrated in AML, where CSC-like leukaemic stem cells are preferentially OXPHOS-dependent (48, 49). Rational co-targeting of both axes — for example, dual 2-DG (HK2/glycolysis inhibition) plus IACS-010759 (Complex I/OXPHOS inhibition) — is therefore required to eliminate both the metabolically active bulk and the OXPHOS-reliant CSC subpopulation simultaneously, preventing metabolic escape and achieving durable responses (49, 50).

#### 2.1.1 Controversies in cancer metabolic biology: contradictory evidence, mechanisms, and context-dependent scenarios

Three interrelated controversies have generated substantial debate in the cancer metabolism field, and their resolution has direct therapeutic implications. First, the glycolysis–OXPHOS dichotomy: the classical Warburg hypothesis posits aerobic glycolysis as the dominant bioenergetic programme of cancer cells. However, mounting evidence demonstrates that OXPHOS is not universally suppressed. In clear cell RCC, pancreatic ductal adenocarcinoma, oesophageal cancer, and castration-resistant prostate cancer, OXPHOS provides the predominant ATP source, and PGC-1α-driven mitochondrial biogenesis actively enhances metabolic fitness rather than diminishing it (48, 50). Critically, within the same tumour mass, distinct cellular compartments simultaneously maintain high glycolytic and high OXPHOS fluxes: hypoxic core cells are HIF-1α-driven glycolytic while well-oxygenated peripheral cells and CSCs rely on OXPHOS. Mechanistically, MYC is the key reconciling factor — unlike HIF-1α, MYC simultaneously induces glycolytic enzymes (GLUT1/3, HK2, LDHA) and mitochondrial biogenesis genes (TFAM, PGC-1α), generating cells that are metabolically hyperactive across both axes. HIF-2α, which stabilises under chronic rather than acute hypoxia, further maintains OXPHOS by cooperating with, rather than antagonising, MYC — a mechanistic distinction from HIF-1α that is clinically exploited by belzutifan in HIF-2α-amplified RCC. The applicable scenario for preferentially glycolytic tumours includes glucose-rich, acutely hypoxic environments where HIF-1α is dominant; OXPHOS-dominant scenarios apply to CSCs, therapy-resistant persister cells, oxygenated tumour niches, and cancers with LKB1 or PTEN loss that constitutively activate mitochondrial metabolism (31, 49).

Second, the context-dependent role of fatty acid oxidation (FAO): FAO was initially considered a suppressed pathway in proliferating cancer cells because *de novo* lipogenesis was assumed dominant. Contradictory findings have since demonstrated that FAO is actively upregulated in specific contexts: nutrient-deprived or matrix-detached conditions, therapy-resistant states, CSC populations, and immune-suppressive cells (Tregs and M2 TAMs) in the TME. In AML and triple-negative breast cancer, FAO provides acetyl-CoA and NADPH for antioxidant defence and supports OXPHOS as an alternative fuel when glycolysis is inhibited. The controversy arises because FAO upregulation can be both pro-tumorigenic (sustaining metabolic flux in stressed cells) and anti-tumorigenic (promoting FAO in immune effector cells that might otherwise be immunosuppressed). Context defines outcome: in nutrient-replete primary tumour bulk, *de novo* lipogenesis dominates; in circulating tumour cells or metastatic niches, FAO is the primary energy source. This duality mandates context-specific biomarker-guided FAO inhibitor deployment rather than universal CPT1 blockade (8, 35).

Third, the compensatory pathway activation controversy: a fundamental mechanistic principle underlying all metabolic therapeutic failures is that cancer cells maintain interconnected, redundant networks capable of rapid compensatory rewiring. When glycolysis is inhibited, cancer cells upregulate OXPHOS via PGC-1α induction. When OXPHOS is blocked, cells activate glycolysis and FAO. When GLS1 is inhibited (CB-839), compensatory pyruvate carboxylation and asparagine biosynthesis sustain TCA anaplerosis. When mTOR is inhibited, ULK1 de-repression drives autophagy that recycles metabolic substrates. This bidirectional metabolic plasticity — governed by AMPK as the energy sensor and HIF-1α and MYC as transcriptional metabolic state switches — represents a fundamental biological barrier that cannot be overcome by sequential monotherapies. The mechanistic resolution is rational combination design: agents targeting complementary metabolic nodes simultaneously (glycolysis + OXPHOS; GLS1 + mTOR; IDH inhibitor + BET bromodomain inhibitor) close the compensatory escape routes that single-agent therapies inevitably open. Applicable scenarios for single-agent metabolic therapy are restricted to tumours with extreme, genetically fixed dependencies such as IDH-mutant 2-HG production (ivosidenib) or VHL-loss-driven HIF-2α amplification (belzutifan), where the genetic driver creates a non-compensatable dependency (27, 28, 51).

### 2.2 TCA cycle, anaplerosis, and oncometabolites

The TCA cycle is a central node for ATP generation and the synthesis of biosynthetic intermediates (e.g., α-KG for epigenetic regulation; succinate, fumarate, malate for gluconeogenesis). Glutamine is the predominant anaplerotic carbon source, entering the TCA cycle via GLS1-mediated conversion to α-KG. Gain-of-function mutations in IDH1/2 produce the oncometabolite 2-hydroxyglutarate (2-HG), which competitively inhibits α-KG-dependent TET demethylases, KDM histone demethylases and PHD prolyl hydroxylases, leading to epigenetically locked differentiation blocks that are resistant to therapy (32, 33). SDH and FH mutations also lead to accumulation of succinate and fumarate, acting as oncometabolites that inhibit the same enzyme family (52). The IDH-mutant epigenetic lock has profound clinical implications: CIMP-positive IDH-mutant lowgrade gliomas exhibit distinct DNA methylation profiles and more indolent trajectories compared to IDH-wild-type glioblastoma. SDH mutations in gastrointestinal stromal tumours and paragangliomas generate succinate accumulation that similarly inhibits TET and PHD enzymes, creating pseudohypoxic states with HIF-1α stabilisation independent of oxygen tension. FH mutations in hereditary leiomyomatosis-associated renal cell carcinoma generate fumarate that covalently modifies cysteine residues (protein succination) in addition to inhibiting α-KG-dependent enzymes. These hereditary oncometabolite syndromes represent natural genetic experiments confirming the causal role of metabolite-driven epigenetic disruption in carcinogenesis.

### 2.3 PPP, redox balance, glutaminolysis, and lipid reprogramming

**Figure 3:**
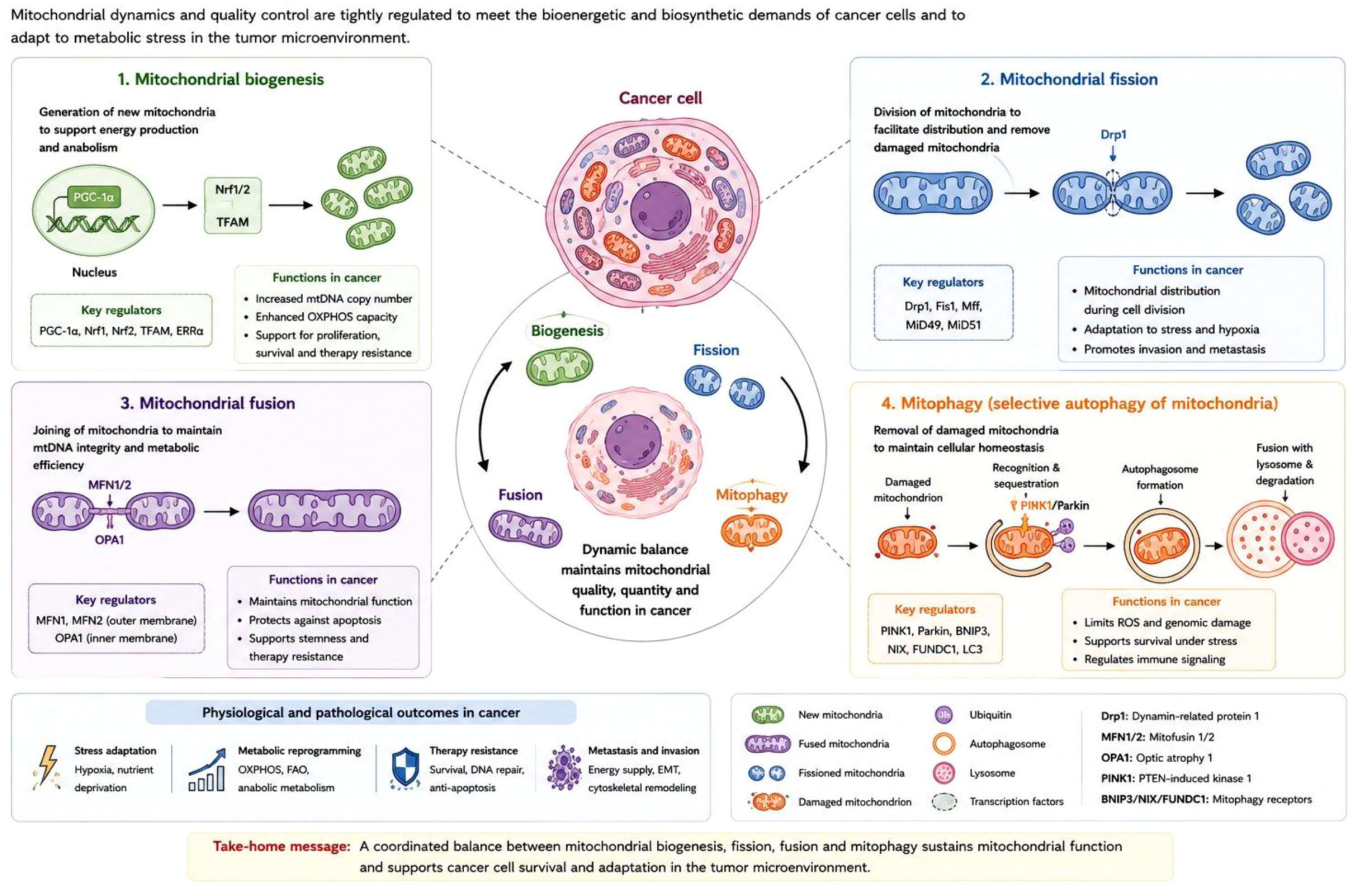
Mitochondrial dynamics in cancer: biogenesis, fission, fusion, and mitophagy. Biogenesis (PGC-1α → NRF1/2 → TFAM) increases OXPHOS capacity and is particularly active in CSCs and OXPHOS-dependent melanoma and castration-resistant prostate cancer; targetable by SR-18292 (PGC-1α inhibitor). Fission (DRP1/Fis1/Mff) facilitates cisplatin resistance via ROS-DRP1 signalling and promotes Warburg-like glycolysis and stemness; targetable by Mdivi-1 (DRP1 GTPase inhibitor). Fusion (MFN1/MFN2, OPA1) maintains mtDNA integrity, optimises ETC supercomplex assembly, and tunes ROS generation at Complexes I and III; OPA1 cristae remodelling regulates cytochrome c release at the apoptotic threshold. Mitophagy (PINK1-Parkin canonical pathway; BNIP3/NIX HIF-1α-driven receptor pathway; FUNDC1 hypoxia-responsive pathway) selectively eliminates depolarised mitochondria, preventing apoptosis under cytotoxic stress and constituting a primary drug resistance mechanism targetable by chloroquine (lysosomal acidification inhibitor). All processes are colour-coded by function: green = pro-survival, red = pro-resistance, blue = therapeutic target. Generated using ChatGPT (OpenAI, 2024).

The PPP provides NADPH (antioxidant defence, reductive biosynthesis) and ribose-5-phosphate (nucleotide synthesis) via G6PD and TKT, both transcriptionally induced by NRF2 and MYC (34). Glutaminolysis beyond TCA anaplerosis supplies nitrogen for nucleotide and amino acid biosynthesis, maintains GSH pools via cystine/glutamate antiporter (system xCT/SLC7A11), and activates mTORC1. One-carbon metabolism via the serine-glycine pathway provides methyl groups for DNA/histone methylation and purine synthesis. *De novo* lipogenesis, driven by the ACLY-ACC-FASN axis and transcriptionally activated by SREBP1/2 downstream of PI3K/AKT/mTOR, supplies membrane phospholipids, lipid signaling molecules and ferroptosis protection via accumulation of lipid droplets (35, 53, 54). FAO supplies NADPH and acetyl-CoA under nutrient stress, especially in therapyresistant and CSC populations (**Figures 3**).

## 3 Transcription factors and molecular regulators

A comprehensive overview of transcription factors and their metabolic targets is provided in **Table 1**. Here we highlight mechanistically critical features and clinically relevant controversies.

### 3.1 HIF-1α and HIF-2α — acute vs. chronic hypoxia adaptation

HIF-1α stabilisation under hypoxia or pseudohypoxia (VHL loss, SDH/FH mutations) induces a comprehensive glycolytic program while simultaneously suppressing TCA entry via PDK1 and enhancing glutaminolysis via reductive carboxylation (1). HIF-2α, structurally similar but functionally distinct, stabilises in chronic hypoxia, maintains OXPHOS, and cooperates with, rather than antagonizes, MYC. This HIF-1α/HIF-2α dichotomy is clinically critical: HIF-2α-amplified RCC and neuroblastoma respond to belzutifan (FDA 2021), while HIF-1α-driven tumours require different targeting strategies (9).

Downstream of HIF-1α, vascular endothelial growth factor (VEGF) is a major transcriptional target driving tumour angiogenesis, enabling oxygen and nutrient delivery to expanding tumour masses. HIF-1α also induces carbonic anhydrase IX (CAIX) to manage acidosis arising from lactate accumulation, a further metabolic adaptation to the hypoxic niche (29). The clinical relevance of HIF-1α-VEGF is evidenced by bevacizumab (anti-VEGF) approval across multiple cancers, and the emerging synergy of anti-angiogenic agents with HIF inhibitors and metabolic blockers.

### 3.2 MYC — master integrator of anabolic metabolism

MYC drives glycolysis (GLUT1/3, HK2, LDHA), glutamine addiction (GLS1 via miR-23a/b suppression), mitochondrial biogenesis (TFAM), nucleotide synthesis (CAD, DHODH), and serine/glycine biosynthesis, making it the master integrator of all anabolic growth programs (13). MYC amplification occurs in >70% of cancers. Although directly undruggable, BET bromodomain inhibitors (JQ1, OTX015, pelabresib) suppress MYC super-enhancer transcription. The combination of BET inhibitors with GLS1 inhibitors, targeting MYC-driven glycolysis and glutamine addiction simultaneously, shows synergistic preclinical efficacy.

### 3.3 p53 — wild-type gatekeeper vs. mutant metabolic driver

Wild-type p53 and gain-of-function mutant p53 exert diametrically opposed metabolic effects, representing a critical context-dependent axis in cancer metabolic reprogramming (36). The mechanistic basis for this functional dichotomy, and its therapeutic implications, are detailed below.

The functional dichotomy between WT and gain-of-function mutant p53 is mechanistically delineated as follows. WT p53 metabolic actions: (1) SCO2 induction enhances cytochrome c oxidase assembly, upregulating OXPHOS; (2) TIGAR induction reduces fructose-2,6-bisphosphate, inhibiting PFK1 and suppressing glycolytic flux; (3) GLS2 induction supports mitochondrial glutamine catabolism and ROS homeostasis; (6) GLUT1/GLUT4 transcriptional repression reduces glucose uptake. Net effect: WT p53 functions as a metabolic tumour suppressor that restores oxidative metabolism and suppresses the Warburg phenotype. In contrast, gain-of-function mutant p53 drives: (1) transcriptional induction of GLUT1 and HK2, amplifying glycolytic flux; (2) SREBP1/2 activation upregulating FASN, ACC, and SCD1 for membrane lipogenesis; (3) mevalonate pathway activation supplying cholesterol and isoprenoids for RAS prenylation and membrane biosynthesis; (6) PPP and NADPH upregulation conferring ROS resistance and multidrug chemoresistance. Net effect: mutp53 acts as an oncometabolic driver amplifying the Warburg phenotype and conferring therapy resistance. These distinct metabolic programmes carry explicit therapeutic implications: APR-246/eprenetapopt reactivates mutp53 to a WT-like conformation; statins exploit mutp53-mevalonate dependency; TVB-2640 targets mutp53-driven lipogenesis via FASN inhibition; and the WT p53-OXPHOS axis suggests vulnerability to OXPHOS inhibitors (IACS-010759) in p53-WT tumours that upregulate mitochondrial respiration as a stress response.

### 3.4 NF-κB, STAT3, NRF2, and lipid-metabolic regulators

NF-κB couples inflammatory TME signals and metabolic rewiring by inducing GLUT1, HK2, PFK and LDHA, generating glycolytic-inflammatory feed-forward loops with particular relevance in obesity-driven cancers (37). As for STAT3, activated by IL-6 and EGF, it uniquely promotes glycolysis and OXPHOS, conferring metabolic plasticity and CSC maintenance (38–40). The protein NRF2 (activated by KEAP1 mutation in ~20% NSCLC) drives G6PD, TKT, and GSH synthesis for comprehensive ROS defense, conferring multidrug resistance (41, 42). SREBPs, hyperactivated by PI3K/AKT/mTOR, transcriptionally drive FASN, ACC, SCD1, HMGCR, and LDLR for membrane biosynthesis and cholesterol metabolism (43). Mutant KRAS enhances glucose uptake and macropinocytosis for amino acid scavenging; KRAS G12C inhibitors (sotorasib, adagrasib) represent the first direct KRAS-targeted therapies (45, 46). Additionally, ERα functions as a metabolic transcription factor in breast cancer, activating GLUT1/glycolytic enzymes and FASN/SCD1 for lipid biosynthesis, with ERα-driven metabolic reprogramming contributing to endocrine therapy resistance (alpelisib plus fulvestrant co-targets this axis); PPAR isoforms (α, β/δ, γ) regulate FAO, lipid uptake, and inflammatory signalling in context-dependent cancer roles (44). Across these regulators, the transcriptional interplay between HIF-1α, MYC, NF-κB, and STAT3 generates integrated metabolic regulatory circuits that amplify oncogenic programmes while simultaneously suppressing anti-tumour immunity — HIF-1α induces glycolytic enzymes in tumour cells while reducing OXPHOS capacity in cytotoxic T cells via lactate-mediated MCT inhibition, and MYC-driven glutamine consumption creates amino acid deprivation zones impairing TCR signalling (24, 55). Targeting multiple nodes of these interconnected circuits simultaneously is therefore essential to overcome metabolic redundancy and achieve durable therapeutic responses.

To synthesise and de-fragment the upstream-downstream crosstalk among these transcription factors and pathways: HIF-1α and MYC form a feedforward loop — HIF-1α transcriptionally induces MYC stabilisers (such as HIF-1α-driven ROS that suppress MYC ubiquitination), while MYC amplifies HIF-1α target gene responsiveness by increasing RNA Pol II loading at hypoxia-responsive elements. Both converge on NF-κB: HIF-1α-driven lactate activates NF-κB via IKKβ phosphorylation, and MYC-driven glutamine depletion triggers NF-κB-mediated stress survival programs. NF-κB in turn activates STAT3 via IL-6 secretion from tumour and stromal cells, creating a paracrine metabolic-inflammatory circuit. STAT3 uniquely bridges glycolysis (GLUT1, HK2) and OXPHOS (via PGC-1α/TFAM co-activation under IL-6 signalling), making it the transcriptional mediator of metabolic plasticity itself. Downstream, STAT3 and MYC both activate SREBP1/2, which drives lipogenesis (FASN, ACC, SCD1) via PI3K/AKT/mTOR — directly connecting growth factor signalling to membrane biosynthesis and ferroptosis protection via lipid droplet accumulation. NRF2, activated downstream of MYC-driven ROS and KRAS/PI3K signalling, closes the antioxidant feedback loop by inducing G6PD and TKT (PPP) to regenerate NADPH consumed by FASN and antioxidant glutathione synthesis. This integrated network — HIF-1α/MYC → NF-κB/STAT3 → SREBP/NRF2 → glycolysis + OXPHOS + lipogenesis + ROS defence — constitutes a self-reinforcing oncometabolic circuit where inhibition of any single node triggers compensatory activation of parallel arms, explaining why multi-target combination strategies are mechanistically required (24, 27, 55) (**Figures 2**, **3**).

## 4 Key metabolic enzymes as effectors and therapeutic targets

Beyond transcriptional control, metabolic enzymes serve as post-translational regulatory nodes and direct therapeutic targets (**Table 3**); key mechanistic features and clinical implications are elaborated below.

**Table 3:** Key metabolic enzymes in cancer: regulation and therapeutic strategies.

Enzyme	Pathway	Cancer role	Regulation	Therapeutic agent	Refs
HK2	Glycolysis (Step 1)	Commits glucose; anti-apoptotic via OMM/VDAC1 interaction	HIF-1α, MYC, PI3K/AKT; G6P allosteric; epigenetic promoter regulation	2-DG; lonidamine; HK2 siRNA nanoparticles	
PKM2	Glycolysis/Nuclear coactivator	Dimeric PKM2 diverts intermediates to biosynthesis; nuclear coactivator for HIF-1α, β-catenin	EGFR/Src phosphorylation (dimer); FBP allosteric (tetramer); acetylation/oxidation PTMs	TEPP-46/ML265; shikonin (PKM2 inhibitor)	
LDH-A	Pyruvate → Lactate	NAD+ regeneration; TME acidification; HIF-1α normoxic stabilisation; immune suppression	MYC, HIF-1α transcription; PCAF acetylation; Src phosphorylation	FX11; GNE-140; LDH-A + ICI (synergy)	
IDH1/2 (mutant)	TCA (neomorphic 2-HG production)	2-HG inhibits TET/KDM/PHD; CpG hypermethylation (CIMP); differentiation block	Gain-of-function mutations (R132H, R140Q, R172K); 2-HG at millimolar levels	Ivosidenib (IDH1, FDA); enasidenib (IDH2, FDA); vorasidenib (IDH1/2, FDA Aug 2024)	
GLS1	Glutaminolysis	TCA anaplerosis; GSH synthesis; mTORC1 activation; MYC-driven glutamine addiction	MYC (miR-23a/b suppression); PI3K/AKT/mTOR; HIF; phosphorylation PTMs	CB-839 (telaglenastat); IPN60090; JHU-083 (DON prodrug)	
FASN/ACLY/G6PD	Lipogenesis; Acetyl-CoA supply; PPP	FASN: membrane biosynthesis, ferroptosis protection. ACLY: TCA-to-lipid link + histone acetylation. G6PD: NADPH, nucleotides, chemo-resistance	FASN: PI3K/AKT/mTOR → SREBP1; mutp53. ACLY: AKT-Ser454. G6PD: NRF2, SIRT2 deacetylation	TVB-2640 (FASN); bempedoic acid (ACLY); 6-AN (G6PD); synthetic lethality with ROS inducers	

References: 53, 54, 56–81.

### 4.1 HK2, PKM2, and LDH-A — glycolytic effectors

HK2 couples glucose commitment to glycolysis with anti-apoptotic signalling via its VDAC1 interaction at the outer mitochondrial membrane, making it a dual metabolic-survival target (56). A mechanistically distinctive feature of cancer metabolism is the PKM2 dimer-tetramer switch: the cancer-predominant dimeric form diverts glycolytic intermediates into biosynthetic pathways and enables nuclear translocation where PKM2 coactivates HIF-1α and β-catenin, creating a feed-forward glycolytic-transcriptional loop; TEPP-46-mediated tetramer enforcement disrupts this nuclear function (60, 61, 63). LDH-A, beyond sustaining glycolytic NAD+ regeneration, exports lactate via MCT4 to acidify the TME and suppress cytotoxic T-cell and NK cell function — a mechanism exploitable by combining LDH-A inhibitors with immune checkpoint blockade to simultaneously restore TIL metabolic fitness and tumour cell killing (64–67).

### 4.2 IDH1/2 — oncometabolite producers

IDH1/2 gain-of-function mutations generate 2-HG, which competitively inhibits TET demethylases (global CpG hypermethylation, CIMP phenotype), KDM histone demethylases (H3K9me3/H3K27me3 accumulation), and PHD prolyl hydroxylases (pseudohypoxic HIF-1α stabilisation). This epigenetic lock blocks differentiation and creates therapy resistance (68). Ivosidenib (IDH1), enasidenib (IDH2), and vorasidenib (brain-penetrant dual IDH1/2, FDA August 2024, INDIGO Phase III, PFS 27.7 vs 11.1 months) act via differentiation therapy by restoring α-KG-dependent epigenetic enzyme function (73).

### 4.3 GLS1, FASN, ACLY, and G6PD

GLS1 executes MYC-driven glutamine addiction; compensatory upregulation of pyruvate carboxylation and FAO upon GLS1 inhibition mandates rational combination with glycolysis inhibitors or mTOR blockade (74, 75). FASN maintains membrane phospholipid synthesis and ferroptosis protection by lipid droplet formation; TVB-2640 is currently in Phase I/II trials for breast, NSCLC, and astrocytoma (78, 79). ACLY links TCA metabolism to cytoplasmic lipogenesis and nuclear histone acetylation via acetyl-CoA supply, positioning it at the epigenetic-metabolic interface (80). G6PD provides NADPH for ROS buffering and nucleotide synthesis; its inhibition combined with ROS-inducing agents creates synthetic lethality in NRF2-high cancers (81).

Post-translational regulation of GLS1 — including SIRT5-mediated desuccinylation (activation) and EGFR phosphorylation at Y394 (stabilisation) — enables rapid metabolic adaptation independent of transcription and represents additional druggable nodes. Downstream of GLS1, glutamate dehydrogenase (GLUD1/2) and aspartate aminotransferase (GOT1/2) route glutamine-derived carbon into TCA and biosynthetic pathways; their combined inhibition with GLS1 blockade achieves more complete glutaminolysis disruption in preclinical pancreatic cancer models (76, 77).

## 5 Mitochondrial dynamics and metabolic plasticity

Mitochondria undergo continuous biogenesis, fission, fusion, and mitophagy that directly regulate metabolic state and therapy resistance. These processes are summarised in **Table 4** (Mitochondrial Dynamics section).

**Table 4:** Epigenetic, mitochondrial, and emerging themes in cancer metabolic reprogramming.

Area	Key component	Mechanism/cancer role	Therapeutic implication	References
Histone Acetylation	Acetyl-CoA/HATs (p300/CBP)/HDACs/Sirtuins	Metabolic state directly controls H3/H4 acetylation at growth gene loci via nuclear acetyl-CoA fluctuations	HDAC inhibitors (vorinostat, romidepsin, FDA-approved); ACLY inhibitors to reduce nuclear acetyl-CoA	
DNA/Histone Methylation	SAM, α-KG, 2-HG, succinate, fumarate/TET enzymes/EZH2 (PRC2)/KDMs	IDH-mutant 2-HG inhibits TET (CpG hypermethylation, CIMP) and KDMs (H3K27me3); SDH/FH mutations create pseudohypoxia	IDH inhibitors restore α-KG-dependent TET/KDM function; tazemetostat (EZH2, FDA-approved); decitabine/5-azacytidine	
Lysine Lactylation	Lactate → lactyl-CoA/CBP/p300 (writer)/Sirtuins (eraser)	H3K18la/H3K9la activate pro-tumour gene programs; TAM M2 polarisation via lactate-driven histone lactylation — direct Warburg-immune link	AZD3965 (MCT1/lactate); CBP/p300 inhibitors; anti-Warburg strategies to reduce TME lactate	
Mitochondrial Dynamics	DRP1 fission; MFN1/2, OPA1 fusion; PINK1-Parkin mitophagy; PGC-1α biogenesis; mtDNA mutations	Fission promotes Warburg/cisplatin resistance; Fusion optimises OXPHOS/ETC supercomplexes; Mitophagy enables therapy resistance; mtDNA Complex I mutations drive glycolytic shift + ICI response↑	Mdivi-1 (DRP1); IACS-010759 (Complex I); chloroquine (mitophagy blockade); PGC-1α inhibitors (SR-18292)	
Emerging Themes	Single-cell/spatial metabolomics; ferroptosis; metabolic ICIs; AI-GSMMs; circadian-metabolism	scMetabolomics resolves TME heterogeneity; spatial MSI maps lactate/tryptophan gradients; GPX4 inhibition triggers immunogenic ferroptosis; AI predicts synthetic lethal metabolic vulnerabilities; BMAL1/CLOCK regulate LDHA, FASN, IDH2	RSL3/erastin (ferroptosis); epacadostat+ICI; AI-driven metabolic patient stratification; chrono-pharmacology dosing optimisation	

References: 8, 63, 68–73, 82–100.

### 5.1 Biogenesis, fission, and fusion

PGC-1α drives mitochondrial biogenesis by coactivating NRF1/2 and TFAM; in OXPHOS-dependent melanoma and castration-resistant prostate cancer, PGC-1α promotes metabolic fitness while in MYC-driven cancers its suppression maintains glycolytic dominance (48, 50, 89, 90, 101). CSCs exhibit PGC-1α-driven enhanced biogenesis and elevated mitochondrial membrane potential, rendering them preferentially sensitive to OXPHOS inhibitors (IACS-010759, metformin) while being resistant to conventional glycolysis-targeted therapy. DRP1-mediated mitochondrial fission promotes Warburg-like glycolysis, cisplatin resistance in ovarian cancer via ROS-DRP1 signalling, and stemness; Mdivi-1 (DRP1 inhibitor) impairs fission-dependent metabolic plasticity preclinically (91). The ETC supercomplex assembly is mediated by MFN1/MFN2 and OPA1-mediated fusion that optimises OXPHOS efficiency; OPA1 remodelling of cristae tunes ROS generation at Complexes I and III (92).

### 5.2 Mitophagy and mtDNA mutations

PINK1-Parkin mediated mitophagy allows the removal of damaged mitochondria to prevent apoptosis and aid cells to survive under cytotoxic stress that is a major mechanism of drug resistance that can be targeted by chloroquine in combination with chemotherapy (93, 96). Critically, autophagy and metabolic reprogramming are mechanistically intertwined in cancer drug resistance: autophagy recycles nutrients and damaged organelles to sustain metabolic flux when canonical pathways are pharmacologically blocked, while metabolic state (AMPK/mTORC1 ratio, NAD+/NADH) determines autophagy induction threshold. Aruchamy et al. (51) comprehensively demonstrated that enhanced glycolysis, OXPHOS, and fatty acid metabolism collectively enable cancer cells to survive therapeutic stress via autophagy-mediated metabolic homeostasis maintenance, establishing autophagy-metabolic crosstalk as a key resistance node (51). HIF-1α induces BNIP3, NIX and FUNDC1 receptor-mediated mitophagy under hypoxia, providing a direct link between hypoxia signalling and mitochondrial quality control. mtDNA mutations in Complex I subunits (particularly Mt-Nd5) induce a Warburg-like glycolytic shift by increasing NADH/NAD+ ratio. Remarkably, a 2024 Nature Cancer study demonstrated that such mutations enhance ICI response in melanoma by remodelling the immune TME, increasing T-cell infiltration—revealing an unexpected metabolic-immunological consequence of mitochondrial dysfunction (95).

Alternative receptor-mediated mitophagy via BNIP3 and NIX, both HIF-1α transcriptional targets—is particularly relevant under hypoxia, where it prevents mitoptosis (massive mitochondrial permeabilisation) and enables cell survival in hypoxic tumour niches. FUNDC1-mediated mitophagy responds to hypoxia and PGAM5-regulated dephosphorylation, representing an additional therapeutic target in hypoxic tumours. The balance between mitophagy as a survival mechanism (pro-resistance) versus mitophagy as a cell death inducer (pro-apoptotic at excessive levels) determines net therapeutic outcome when combining mitophagy modulators with standard cytotoxics. Preclinical optimisation of chloroquine dosing to achieve mitophagy blockade without complete lysosomal shutdown is an active research priority (94).

## 6 Epigenetic regulation of cancer metabolism

Metabolism and epigenetics form a bidirectional regulatory axis: metabolic intermediates serve as cofactors for chromatin-modifying enzymes, while epigenetic programs control metabolic gene expression. Key layers are summarised in **Table 4** (Epigenetics section).

### 6.1 Acetyl-CoA, histone acetylation, and HDAC biology

Nuclear acetyl-CoA concentrations, supplied by glycolysis (via pyruvate dehydrogenase and ACLY-mediated citrate cleavage), FAO, and acetate (via ACSS2), directly control global H3/H4 acetylation patterns at growth gene promoters and enhancers (82). Under glucose abundance, elevated acetyl-CoA boosts H3K9ac and H3K27ac at oncogenic super-enhancers; under nutrient restriction, ACSS2-driven local acetyl-CoA provision maintains gene-specific acetylation. HDAC inhibitors (vorinostat, romidepsin, FDA-approved for CTCL) exploit this metabolic-epigenetic dependency; combination with ACLY or glycolysis inhibitors is under preclinical investigation (83).

### 6.2 SAM, α-KG, 2-HG, and the methylation axis

SAM (methionine cycle product) is the universal methyl donor for DNMT and PMT enzymes; α-KG is the essential cofactor for TET DNA demethylases and JmjC KDM histone demethylases. IDH1/2-mutant 2-HG, and SDH/FH mutation-derived succinate/fumarate, competitively inhibit both TET and KDM enzymes, causing genome-wide CpG hypermethylation and H3K27me3 accumulation that epigenetically silence tumour suppressors and differentiation genes (84, 85). IDH inhibitors (ivosidenib, enasidenib, vorasidenib) restore α-KG-dependent TET/KDM function. EZH2 (PRC2 catalytic subunit) depositing repressive H3K27me3 is targetable by tazemetostat (FDA-approved for epithelioid sarcoma and follicular lymphoma) (85, 86).

### 6.3 Lysine lactylation — a novel warburg-epigenetic coupling

Lysine lactylation, where lactate-derived lactyl-CoA modifies histone lysines (H3K18la, H3K9la) via CBP/p300, is a recently discovered post-translational modification that directly couples aerobic glycolysis to epigenetic gene regulation (63). In hypoxic, glycolytic TME niches, histone lactylation activates pro-tumour wound-healing and immune evasion programs. In TAMs, tumour-exported lactate drives H3K18la and M2-like polarization, directly connecting the Warburg effect to immunosuppressive. TME remodelling. AZD3965 (MCT1 inhibitor) and CBP/p300 inhibitors represent dual metabolic-epigenetic intervention strategies targeting this axis (88).

Non-coding RNAs constitute a dynamic post-transcriptional regulatory layer governing cancer metabolism. Oncogenic microRNAs (miR-21, miR-155) silence PTEN and metabolic tumour suppressors, activating PI3K/AKT/mTOR-driven anabolism, while tumour-suppressive miR-23a/b (targeting GLS1) and miR-122 (targeting LDHA/PKM2) are frequently downregulated by promoter hypermethylation. Long non-coding RNAs including HOTAIR, MALAT1, and LINC00092 regulate chromatin remodelling and metabolic gene splicing—LINC00092 enhances PFKFB2-driven glycolysis while HOTAIR globally reprogrammes PRC2-dependent chromatin accessibility at metabolic gene loci (84). BET bromodomain inhibitors (JQ1, OTX015) targeting BRD4 at MYC and FASN super-enhancers represent a direct metabolic-epigenetic therapeutic convergence.

Sirtuins (SIRT1–7) are NAD+-dependent class III HDACs that sense the NAD+/NADH ratio, a direct indicator of metabolic redox state, and translate metabolic status into epigenetic regulation (84). SIRT1 deacetylates and activates PGC-1α (mitochondrial biogenesis), FOXO transcription factors, and p53 while destabilising HIF-1α under normoxia. SIRT3 (mitochondrial) activates OXPHOS complex subunits, IDH2, and MnSOD via deacetylation. SIRT6 inhibits glycolysis by deacetylating H3K9ac at HIF-1α target gene promoters and thereby restricting GLUT1 and LDHA expression. Cancer cells are prone to suppress SIRT3 and SIRT6 to sustain glycolytic and lipogenic pathways, leading to a dependence on NAD+ biosynthesis enzyme (NAMPT) that can be exploited therapeutically.

## 7 Immune system and tumour microenvironment: metabolic crosstalk

**Figure 4:**
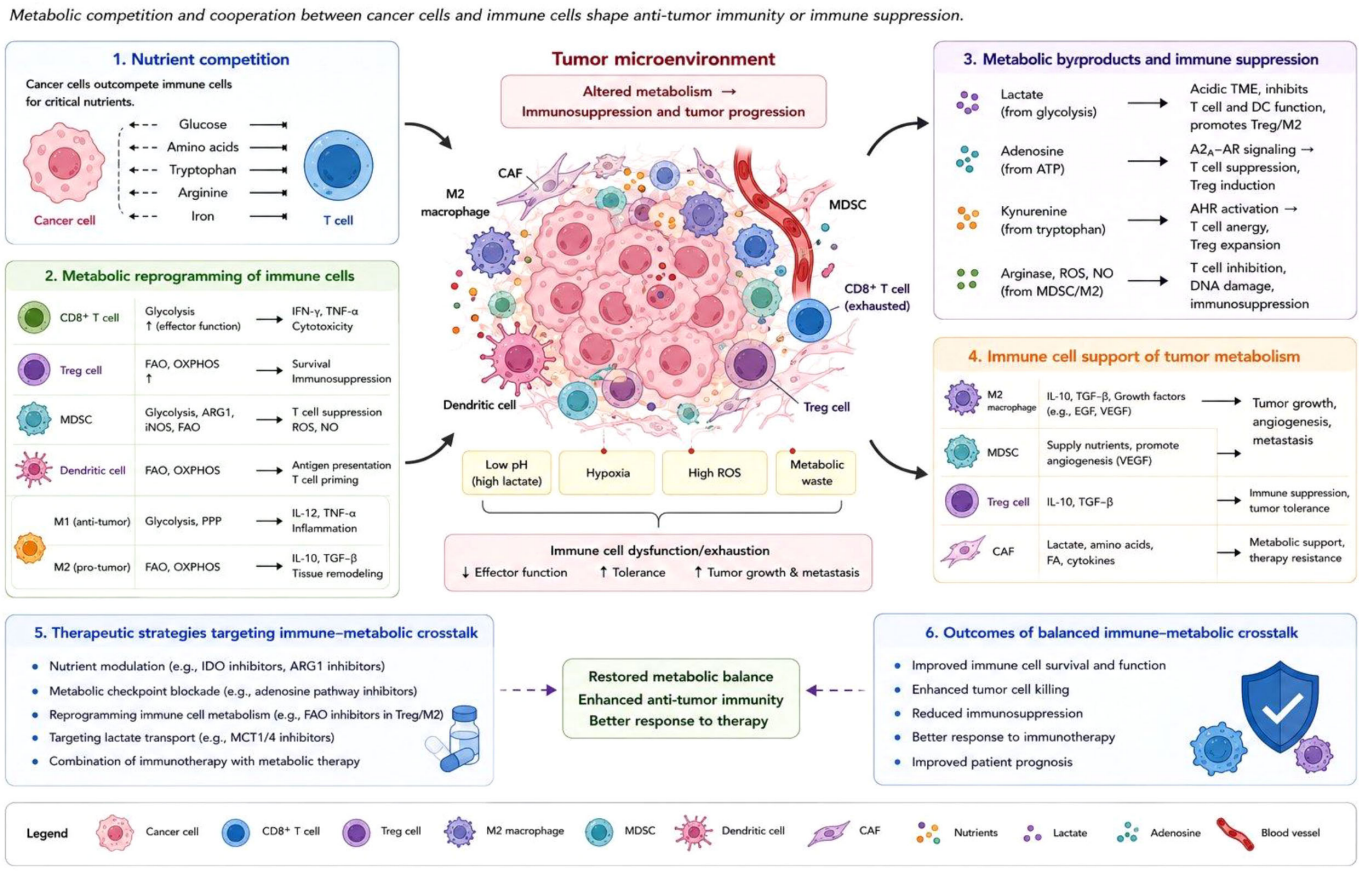
Immune-metabolic crosstalk in the tumour microenvironment (TME). The figure illustrates the metabolically hostile TME niche characterised by glucose and amino acid depletion, lactate and proton accumulation, lipid excess, and hypoxia. Central panel: nutrient competition between glycolytic cancer cells and anti-tumour immune effectors (CD8+ T cells, NK cells). Surrounding panels: metabolic phenotypes of key immune populations — CD8+ T cells (glycolysis for activation; OXPHOS/FAO for memory; exhaustion driven by glucose deprivation and lactate-mediated GPR81-independent signalling); Tregs (FAO/OXPHOS-dominant; thrive in nutrient-depleted TME; kynurenine/AhR axis drives immunosuppressive gene programme); TAMs (M1-like: glycolytic, pro-inflammatory; M2-like: OXPHOS/FAO, immunosuppressive; M2 polarisation driven by lactate-mediated H3K18la histone lactylation); CAFs (reverse Warburg phenotype: glycolytic CAFs secrete lactate/pyruvate consumed by adjacent OXPHOS-competent cancer cells via MCT1). Bottom panel: immunosuppressive metabolic byproducts — lactate (T/NK cell inhibition), adenosine (CD39/CD73 axis, A2A receptor-mediated suppression), kynurenine (IDO1/TDO2-generated, AhR activation promoting Treg differentiation), and arginase-1 (arginine depletion reducing CD3ζ expression on T cells). Therapeutic interventions annotated at each node: epacadostat (IDO1), INCB001158 (arginase), AZD4635 (A2AR), AZD3965 (MCT1), anti-CSF1R (TAM depletion). Generated using ChatGPT (OpenAI, 2024).

The TME is a metabolically hostile niche, glucose/amino acid depletion, lactate/proton accumulation, lipid excess, hypoxia, that simultaneously fuels tumour cells and impairs anti-tumour immunity. Immune-metabolic interactions are summarised in **Table 4** and **Figure 4**.

### 7.1 T cell immunometabolism and exhaustion

Effector CD8+ T cells depend on glycolysis for rapid activation and biosynthesis; memory T cells rely on OXPHOS and FAO for long-term persistence. In the TME, Warburg-active tumour cells compete for glucose and glutamine, which restricts TIL metabolic capacity, favoring exhaustion transcriptional programs (24). Lactate directly inhibits T-cell glycolysis by GPR81-independent mechanisms and induces the exhaustion epigenome. ICI-mediated PD-1 blockade metabolically rescues TIL glucose uptake and mitochondrial function. Importantly, OXPHOS-enhanced CAR-T cells, engineered by PGC-1α overexpression or ex vivo metabolic conditioning, demonstrate superior persistence, memory formation, and anti-tumour efficacy in preclinical and early clinical models.

T cell receptor (TCR) signalling itself modulates metabolic reprogramming: CD28 co-stimulation activates PI3K/AKT/mTOR to promote glycolytic switching for effector function, while CTLA-4 reverses this metabolic activation by competing for CD28 ligand. This explains why CTLA-4 blockade (ipilimumab) metabolically rescues effector T cells beyond its classical PD-1-independent mechanism. Memory T cell formation requires a metabolic transition from glycolysis to OXPHOS/FAO, governed by AMPK activation, PGC-1α upregulation, and mTORC1 suppression. Tumour-induced metabolic exhaustion, characterised by elevated mitochondrial ROS, reduced oxidative capacity, and increased fatty acid oxidation—corresponds to the epigenetic exhaustion program marked by TOX expression and is potentially reversible by OXPHOS-enhancing interventions (58). Regulatory T cells (Tregs) thrive in the metabolically depleted TME by preferentially using FAO and OXPHOS rather than glycolysis, exploiting kynurenine (IDO1-generated) via AhR for immunosuppressive program maintenance. Adipose tissue-rich TMEs (omentum, breast fat pad) further advantage Tregs and M2 TAMs by providing abundant fatty acid substrates for FAO-driven immunosuppressive metabolism. This adipose-immune metabolic axis is particularly relevant in obesity-associated cancers and supports the clinical investigation of FAO inhibitors (etomoxir, CPT1 inhibitors) to selectively impair immunosuppressive cells within the TME (59).

### 7.2 TAMs, CAFs, NK cells, and DCs — metabolic symbiosis and suppression

TAMs exist along an M1 (glycolytic, pro-inflammatory) to M2 (OXPHOS/FAO, immunosuppressive) spectrum. Lactate-driven histone lactylation promotes M2 polarisation in TAMs, mechanistically linking Warburg metabolism to immunosuppression. Rational TME metabolic strategies include inhibition of MCT4 to decrease lactate export, blockade of CSF1R to deplete TAMs and supplementation with succinate to promote M1 repolarisation (62, 87). CAFs adopt the ‘reverse Warburg effect’ phenotype a glycolytic reverse Warburg phenotype, secreting lactate and pyruvate imported by adjacent OXPHOS-competent cancer cells via MCT1, a spatial metabolic symbiosis. NK cells depend on OXPHOS/mTOR-driven IL-15 signalling; IDO1-generated kynurenine and lipid overloading in the TME suppress NK cytotoxicity. DCs need FAO for antigen cross-presentation; CD36-mediated oxidised lipid uptake impairs DC-primed CD8+ T cell responses – a lipid-immune checkpoint. CSCs have an OXPHOS-dominant, immunologically quiescent metabolism shielded by lipid droplets, enabling therapy and immune escape.

### 7.3 Metabolic immune checkpoints

Beyond PD-1/CTLA-4 protein checkpoints, cancer cells exploit enzymatic metabolic immune checkpoints. IDO1/TDO2 catabolise tryptophan to kynurenine, activating AhR in T cells, promoting exhaustion and Treg differentiation (102). Arginase-1 in myeloid cells depletes arginine critical for T-cell CD3ζ expression (103). CD39/CD73-generated extracellular adenosine suppresses NK and T cells via A2A receptor signalling (104). IDO1 inhibitors (epacadostat, BMS-986205), arginase inhibitors (INCB001158), and A2A antagonists (AZD4635) are in Phase I/II trials in combination with anti-PD-1, with ongoing patient selection refinement.

## 8 Therapeutic implications and metabolic targeting strategies

**Figure 5:**
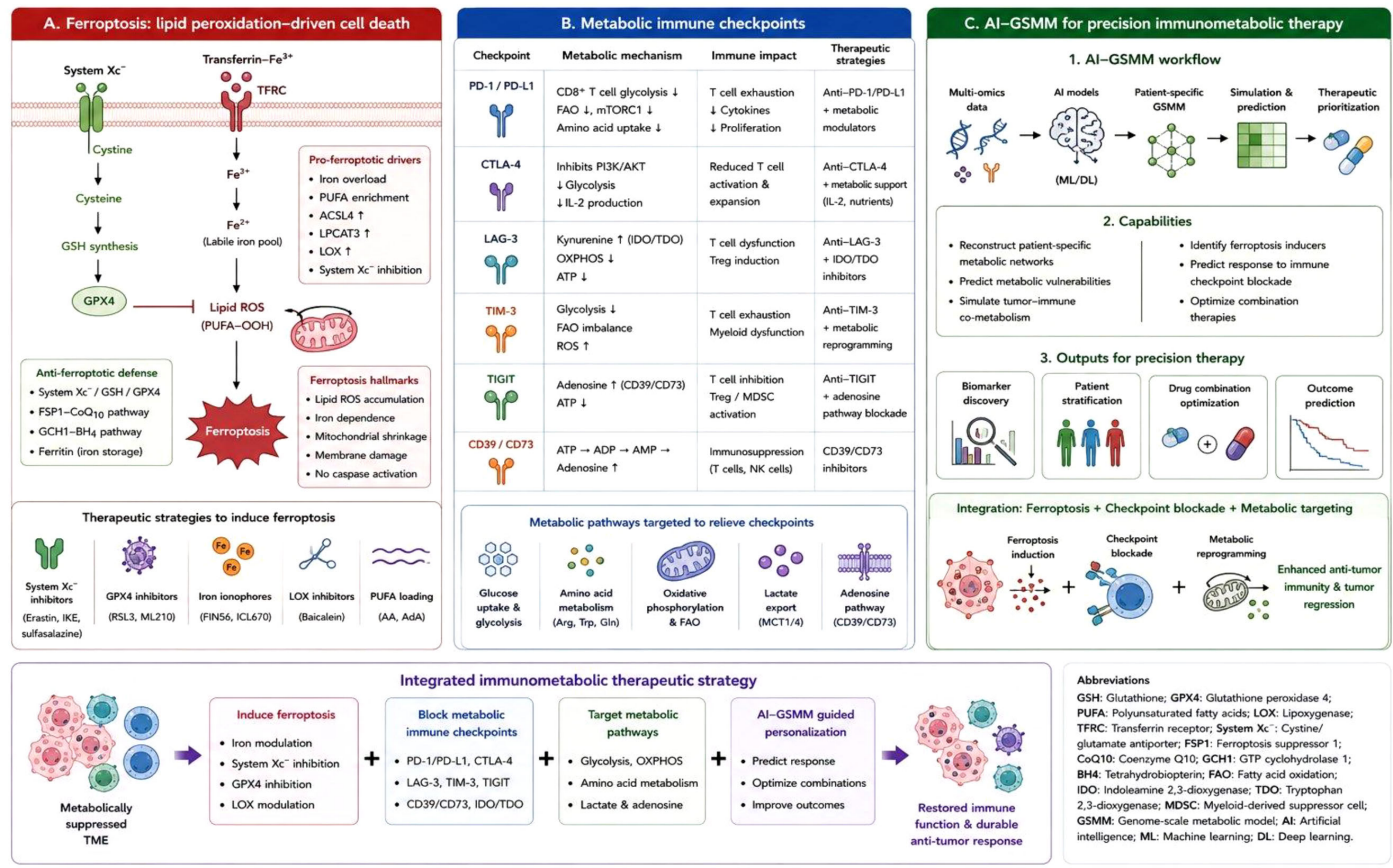
Ferroptosis, metabolic immune checkpoints, and AI genome-scale metabolic modelling (GSMM) for precision immunometabolic therapy. **(A)** Ferroptosis pathway: GPX4 inactivation triggers lipid peroxide accumulation (PUFA-OOH); system xCT/SLC7A11 mediates cystine import for GSH synthesis; CD8+ T cell-derived IFN-γ downregulates SLC7A11, sensitising tumour cells to ferroptosis (immune-ferroptosis synthetic lethality); immunosuppressive cytokines (IL-4, IL-10, TGF-β) upregulate GPX4/SLC7A11 conferring ferroptosis resistance; therapeutic inducers annotated: RSL3/ML162 (direct GPX4 inhibitors), erastin (system xCT inhibitor), iron ionophores; DAMPs released (HMGB1, calreticulin) stimulate anti-tumour immunity — positioning ferroptosis inducers as immunogenic cell death agents synergistic with ICI. **(B)** Metabolic immune checkpoints beyond PD-1/CTLA-4: IDO1/TDO2 (tryptophan → kynurenine → AhR/T-cell exhaustion/Treg induction); Arginase-1/2 (arginine depletion/CD3ζ loss); CD39/CD73/A2A adenosine axis (ATP → AMP → adenosine → T-cell/NK suppression); therapeutic agents at each node: epacadostat (IDO1), BMS-986205 (IDO1), INCB001158 (arginase), AZD4635 (A2AR). **(C)** AI-GSMM workflow: multi-omics input (transcriptomics, proteomics, metabolomics) → patient-specific genome-scale metabolic model construction → flux balance analysis identifies synthetic lethal metabolic vulnerabilities → deep learning metabolic subtype classifier for clinical trial stratification → patient-derived organoid validation. Generated using ChatGPT (OpenAI, 2024).

Therapeutic agents targeting cancer metabolic reprogramming are summarized in **Table 5**; **Figure 5**.

**Table 5:** Approved and investigational agents targeting cancer metabolic reprogramming.

Target/pathway	Drug	Mechanism	Cancer type	Status	Refs
HIF-2α	Belzutifan	Selective HIF-2α PAS-B antagonist; blocks HIF-α/β dimerization	RCC, VHL disease, glioblastoma	FDA-approved 2021	
IDH1 (mutant)	Ivosidenib	Suppresses 2-HG; restores TET/KDM function; differentiation therapy	AML, cholangiocarcinoma	FDA-approved 2018/2021	
IDH2 (mutant)	Enasidenib	IDH2-specific 2-HG suppression; myeloid differentiation	AML	FDA-approved 2017	
IDH1/2 (mutant)	Vorasidenib	Brain-penetrant dual IDH1/2 inhibitor; INDIGO Phase III (PFS 27.7 vs 11.1 mo)	Grade 2 IDH-mutant glioma	FDA-approved Aug 2024	
mTORC1	Everolimus/Temsirolimus	Rapalogs; inhibit SREBP-driven lipogenesis, anabolic metabolism	RCC, PNET, HR+/HER2− breast cancer	FDA-approved	
MCT1 (lactate transport)	AZD3965	MCT1 inhibition; disrupts glycolytic-oxidative lactate symbiosis in TME	DLBCL, SCLC, breast cancer	Phase I/II (NCT01791595)	
GLS1	CB-839 (telaglenastat)	Selective GLS1 inhibition; blocks glutamine catabolism and TCA anaplerosis	TNBC, RCC, AML (combinations)	Phase I/II	
FASN	TVB-2640	FASN inhibition; disrupts *de novo* fatty acid synthesis and membrane biosynthesis	Breast, NSCLC, astrocytoma	Phase I/II	
Complex I/OXPHOS	IACS-010759; Metformin	Mitochondrial respiration inhibition; targets OXPHOS-dependent cancer cells	AML, GBM (NCT03291938)	Phase I	
IDO1/Metabolic ICI	Epacadostat; INCB001158 (arginase); AZD4635 (A2AR)	IDO1: suppress kynurenine/AhR axis. Arginase: restore arginine for T cells. A2AR: adenosine blockade	Multiple solid tumours (combinations with anti-PD-1/PD-L1)	Phase I/II	

References: 2, 9–12, 15–17, 48–50, 68–79, 102–106.

### 8.1 Approved metabolic therapeutics and differentiation therapy

IDH inhibitors are the paradigm-defining success of cancer metabolic therapy. Ivosidenib (IDH1) received FDA approval for AML (2018) (69) and IDH1-mutant cholangiocarcinoma (2021, ClarIDHy trial (70)). Enasidenib (IDH2) was approved for AML (2017) (71, 72). The landmark INDIGO Phase III trial established vorasidenib, a brain-penetrant dual IDH1/2 inhibitor—as the first targeted therapy for grade 2 IDH-mutant glioma (PFS: 27.7 vs 11.1 months), receiving FDA approval in August 2024 (73). Belzutifan (HIF-2α antagonist), mTOR rapalogs (everolimus, temsirolimus), and KRAS G12C inhibitors (sotorasib, adagrasib) complete the landscape of approved cancer metabolic therapeutics.

The mechanistic basis for IDH inhibitor efficacy, differentiation therapy rather than direct cytotoxicity, established a new therapeutic paradigm for metabolically targeted cancer treatment analogous to all-trans retinoic acid (ATRA) in acute promyelocytic leukaemia. A key challenge is the emergence of resistance via IDH isoform switching (IDH1-mutant tumours becoming IDH2-mutant upon ivosidenib pressure), second-site IDH mutations disrupting inhibitor binding, or bypass resistance via epigenetic rewiring independent of 2-HG. Combination strategies pairing IDH inhibitors with BET inhibitors (to suppress MYC-driven metabolic bypass), DNMT inhibitors (to reinforce demethylation), or immune checkpoint blockade (to exploit differentiation-driven neoantigen exposure) are under active clinical evaluation to improve durable response rates (73).

Adaptive resistance remains the principal challenge for all metabolic therapeutics. For IDH inhibitors, resistance mechanisms include isoform switching (IDH1-mutant AML acquiring IDH2 mutations under ivosidenib pressure), second-site IDH mutations disrupting inhibitor binding (S280F in IDH1), and epigenetic bypass via MYC amplification or RAS pathway activation restoring proliferative signalling independent of 2-HG. For mTOR rapalogs, resistance arises via 4EBP1 hyperphosphorylation bypassing S6K feedback, PI3K pathway reactivation through IRS-1, and autophagy induction restoring metabolic flux. These resistance mechanisms dictate rational salvage strategies: IDH isoform co-inhibitors, mTOR kinase inhibitors (vs rapalogs), and combination with metabolic pathway blockers that prevent compensatory rewiring (15–17, 73). The central mechanistic framework for understanding these resistance patterns is metabolic plasticity: both IDH and mTOR resistance share the common thread of dynamic metabolic rewiring that bypasses target inhibition rather than simple genetic resistance. For IDH inhibitors, resistance is fundamentally oncometabolite-independent epigenetic rewiring — tumours can restore malignant epigenetic programs via MYC-driven or RAS-driven mechanisms even when 2-HG is successfully suppressed. Rational combination strategies accordingly pair IDH inhibitors with BET bromodomain inhibitors (to suppress MYC super-enhancer-driven metabolic bypass), DNMT inhibitors (to reinforce epigenetic demethylation), or immune checkpoint blockade (to exploit differentiation-driven neoantigen exposure). For mTOR rapalogs, resistance is driven by autophagy-mediated metabolic flux restoration — whereby mTORC1 inhibition de-represses ULK1, inducing autophagy that recycles metabolic substrates and sustains anabolic biosynthesis independently of mTOR activity (51). Co-targeting autophagy (chloroquine, hydroxychloroquine) with mTOR inhibitors, or using next-generation mTOR kinase inhibitors (rather than allosteric rapalogs) to achieve complete 4EBP1 dephosphorylation, represent mechanistically grounded salvage approaches.

#### 8.1.1 Extended drug resistance analysis: belzutifan, KRAS inhibitors, GLS1 inhibitors, and MCT1 inhibitors

Beyond IDH inhibitors and mTOR rapalogs, resistance mechanisms have been characterised for all major approved and investigational metabolic agents, and metabolic plasticity is the unifying conceptual framework across all cases. For belzutifan (HIF-2α antagonist, FDA-approved 2021 for RCC/VHL disease), the primary resistance mechanism is HIF-1α compensatory upregulation: when HIF-2α is pharmacologically blocked, tumour cells with retained HIF-1α signalling capacity restore glycolytic gene expression (GLUT1, HK2, LDHA) and angiogenic VEGF production via HIF-1α, bypassing HIF-2α dependency. Secondary resistance arises from acquisition of VHL secondary mutations that reduce belzutifan binding affinity, and from mTORC1-driven SREBP1 activation sustaining lipogenesis independently of HIF-2α. The rational salvage strategy pairs belzutifan with mTOR inhibitors (to block SREBP1-driven lipogenesis) or HIF-1α inhibitors (acriflavine, PX-478), and combination with everolimus is under clinical evaluation in Phase I/II trials (9, 50).

For KRAS G12C inhibitors (sotorasib, adagrasib), resistance is particularly rapid, occurring within median 6–9 months. Metabolic resistance mechanisms include: (i) KRAS-independent reactivation of RAS-MAPK-driven glycolysis via KRAS G12V/D secondary mutations or RTK bypass (EGFR, MET, FGFR amplification) restoring PI3K/AKT/mTOR-driven anabolism independently of KRAS G12C; (ii) metabolic reprogramming toward FAO and OXPHOS in KRAS-inhibited cells that reduce glycolytic dependency, creating a KRAS-independent metabolic persister state targetable by IACS-010759 or metformin; and (iii) macropinocytosis suppression upon KRAS inhibition paradoxically enhancing glutamine dependency via upregulated GLS1, providing a synthetic lethal vulnerability for CB-839 combination with sotorasib/adagrasib. The metabolic plasticity principle is most starkly illustrated here: KRAS inhibition does not abolish cell-autonomous energy production but redirects fuel dependency, mandating concurrent metabolic pathway co-targeting (28, 45, 46).

For GLS1 inhibitors (CB-839/telaglenastat), intrinsic resistance is driven by metabolic pathway redundancy: cells with high pyruvate carboxylase (PC) activity divert glucose-derived pyruvate directly into the TCA cycle via oxaloacetate, bypassing GLS1-dependent glutamine anaplerosis entirely. Acquired resistance involves transcriptional upregulation of asparagine synthetase (ASNS), which uses aspartate to produce asparagine for nucleotide synthesis independently of glutaminolysis. Additionally, FAO-driven acetyl-CoA sustains TCA cycle function under GLS1 blockade, particularly in CSC populations. These mechanisms explain the modest single-agent activity of CB-839 in Phase I/II trials and motivate combination strategies that show synergy: CB-839 + everolimus (RCC; mTOR inhibition blocks PC induction), CB-839 + pembrolizumab (multiple solid tumours), and CB-839 + azacitidine (AML; epigenetic-metabolic combination). The metabolic plasticity insight is that GLS1 inhibition is most effective as a sensitising agent that narrows the metabolic escape routes when combined with orthogonal blockade (74–76).

For AZD3965 (MCT1 inhibitor, NCT01791595), the primary resistance mechanism is MCT4 upregulation: cancer cells that increase MCT4 expression can export lactate via MCT4, bypassing MCT1 blockade and maintaining glycolytic flux. MCT4 expression level is therefore a predictive biomarker of intrinsic resistance, and combination with MCT4 inhibitors (in development) or LDH-A inhibitors (to reduce lactate production at source) addresses this escape route. A secondary mechanism is the metabolic shift from glycolytic symbiosis to autonomous OXPHOS in MCT1-blocked cells that retain mitochondrial capacity, maintaining energy independence without requiring lactate import. The rational applicable scenario for AZD3965 is therefore tumours with documented low baseline MCT4 expression, high glycolytic-oxidative lactate symbiosis between tumour cell subpopulations, and limited mitochondrial reserve — a phenotype assessable by [18F]-FDG PET combined with SCENITH metabolic profiling. Taken together, across all approved and investigational metabolic agents, resistance converges on two universal mechanisms rooted in metabolic plasticity: (i) compensatory pathway switching (glycolysis ↔ OXPHOS ↔ FAO ↔ glutaminolysis), and (ii) transcriptional reprogramming that re-establishes fuel flux through alternative routes. Overcoming this requires combination strategies that simultaneously block multiple metabolic nodes, and patient selection based on direct metabolic flux phenotyping rather than genomic proxies alone (8, 105, 106).

### 8.2 Emerging candidates and combination strategies

AZD3965 (MCT1 inhibitor, NCT01791595) (105, 106) breaks glycolytic-oxidative lactate symbiosis; MCT4 expression predicts resistance. CB-839 (telaglenastat, GLS1) is synergistic with everolimus (RCC), pembrolizumab and EGFR inhibitors in Phase I/II trials. IACS-010759 (Complex I inhibitor, NCT03291938) targets OXPHOS-dependent AML and GBM but dose-limiting neurotoxicity needs refinement of patient stratification (49). The key challenge is metabolic adaptive resistance: glycolysis inhibition induces OXPHOS; OXPHOS inhibition activates glycolysis/FAO; GLS1 inhibition induces pyruvate carboxylation. Rational combination strategies targeting multiple interconnected metabolic nodes, dual glycolysis + OXPHOS blockade; GLS1 + mTOR; IDH inhibitor + hypomethylating agents (AGILE trial, ivosidenib + azacitidine in AML), preempt this compensatory rewiring. Integrating metabolic inhibitors with ICI is particularly compelling: metabolic normalisation of the TME restores TIL glucose availability and reverses exhaustion while simultaneously reducing immunosuppressive metabolite production.

## 9 Emerging themes in cancer metabolic biology

Key emerging areas and their technologies are summarized in **Table 4**.

### 9.1 Single-cell and spatial metabolomics

Bulk metabolomics masks intra-tumoral metabolic mosaicism. Single-cell metabolomics (CE-MS, nanoLC-MS, SCENITH by flow cytometry, live-cell ¹³C tracing in microfluidics) resolves cell-to-cell differences in glycolytic, TCA, and redox fluxes—identifying drug-tolerant persister metabolic states and lineage-specific metabolic wiring (8, 107). Spatial metabolomics/imaging mass spectrometry (MALDI-MSI, DESI-MSI) maps metabolite distributions at 10–50 μm resolution on tissue sections; correlative Visium/GeoMx/CODEX spatial multi-omics assigns metabolic niches to specific cellular compartments. Key discoveries include sharp lactate/acidosis gradients suppressing cytotoxic TILs near hypoxic tumour cores, tryptophan/arginine depletion zones creating T-cell exhaustion niches, and invasive front FAO enrichment driving EMT. Emerging spatial fluxomics overlays ¹³C tracer enrichment onto tissue sections for *in situ* pathway flux resolution (85).

### 9.2 Ferroptosis, AI-driven discovery, and circadian biology

Ferroptosis, an iron-dependent form of regulated cell death, is induced by inactivation of GPX4 and lipid peroxidation and represents a novel metabolic vulnerability (97). OXPHOS-high cells are preferentially ferroptosis-sensitive due to elevated mitochondrial ROS. Ferroptotic cells release DAMPs (HMGB1, calreticulin) stimulating anti-tumour immunity, positioning GPX4 inhibitors (RSL3, ML162) and system xCT inhibitors (erastin) as immunogenic cell death inducers synergistic with ICI. AI-driven genome-scale metabolic models (GSMMs) integrated with patient multi-omics computationally predict synthetic lethal metabolic vulnerabilities specific to individual tumour metabolic wiring, enabling personalised drug target prioritisation (98). Deep learning metabolic subtype classifiers are revealing pan-cancer metabolic stratification systems for clinical trial enrichment. The circadian clock (CLOCK/BMAL1) directly controls expression of LDHA, FASN, IDH2, and OXPHOS components; clock gene disruption accelerates Warburg phenotype and impairs anti-tumour immunity (99). Chrono-pharmacological dosing of metabolic inhibitors aligned to circadian windows of maximal target expression shows preclinical efficacy improvements (100).

Multi-omics data integration for GSMM construction requires careful normalisation across transcriptomic, proteomic, and metabolomic platforms; single-cell multi-omics (scRNA-seq + scATAC-seq + metabolomics) is advancing the field toward single-cell GSMM resolution. Graph neural networks applied to metabolic reaction networks can predict context-specific flux distributions and identify bottleneck reactions exploitable as synthetic lethal targets. The application of these AI frameworks to patient-derived organoid metabolomics, capturing ex vivo tumour metabolic heterogeneity, is enabling rapid, patient-specific metabolic drug sensitivity prediction that may soon inform real-time clinical decision-making (99).

The ferroptosis-immunotherapy intersection is mechanistically grounded in the observation that GPX4 activity is regulated by CD8+ T cell-secreted interferon-gamma (IFN-γ), which downregulates SLC7A11/xCT expression on tumour cells, reducing cystine import and GSH synthesis—thereby sensitising tumour cells to ferroptosis. This IFN-γ-ferroptosis axis means that T cell activation itself primes tumours for lipid peroxidation-mediated death, and GPX4 inhibitors amplify this immune-metabolic synthetic lethality. Conversely, immunosuppressive TME cytokines (IL-4, IL-10) and TGF-β upregulate GPX4 and SLC7A11, conferring ferroptosis resistance. Disrupting the immunosuppressive cytokine milieu that protects tumour cells from ferroptosis represents a novel combination immunotherapy-ferroptosis strategy (88).

Gut microbiome metabolites, including short-chain fatty acids (SCFAs: butyrate, propionate, acetate), bile acid derivatives and tryptophan metabolites (indoles), profoundly impact on tumour metabolic reprogramming and anti-tumour immunity. Butyrate inhibits HDAC activity in colonic epithelial cells and is an epigenetic tumour suppressor, and loss of butyrate-producing Firmicutes is linked to risk of colorectal cancer and non-response to ICIs. Secondary bile acids activate FXR and TGR5 receptors on immune and tumour cells, thus modulating lipid metabolism and inflammatory signalling. The microbiome-immunity-metabolism axis is emerging as a rational target for metabolic probiotic supplementation, faecal microbiome transplantation and precision dietary interventions to enhance ICI efficacy and metabolic therapy outcomes.

### 9.3 Metabolic heterogeneity across tumour compartments: cancer stem cells, primary tumour bulk, and metastatic lesions

Tumour metabolic heterogeneity encompasses distinct metabolic phenotypes operating simultaneously within a single tumour. **Table 6** provides a structured comparative analysis across three key compartments. Cancer stem cells (CSCs) are predominantly OXPHOS-dependent, driven by PGC-1α-mediated mitochondrial biogenesis, and are shielded by lipid droplet accumulation that confers ferroptosis resistance and immune quiescence. This OXPHOS-dominant phenotype renders CSCs resistant to glycolysis-targeted agents (2-DG, lonidamine) while conferring sensitivity to OXPHOS inhibitors (IACS-010759, metformin) — a clinically actionable metabolic vulnerability. Primary tumour bulk exhibits spatial metabolic heterogeneity: hypoxic cores are HIF-1α-driven and glycolysis-dominant, while oxygenated peripheries maintain OXPHOS capacity and greater metabolic plasticity. This spatial metabolic gradient means that biopsies from different tumour regions may yield discordant metabolic phenotype data, complicating biomarker-guided therapy. Metastatic lesions demonstrate distinct metabolic adaptations: circulating tumour cells rely on FAO for energy independence during oxidative stress in the bloodstream; mesenchymal-state metastatic cells show elevated glutaminolysis at secondary sites; and organ-specific metabolic symbiosis at metastatic niches (e.g., lipid-rich omental metastasis in ovarian cancer favouring FAO) dictates site-specific metabolic dependencies distinct from the primary tumour. Understanding these compartment-specific metabolic programmes is essential for designing rational combination strategies that simultaneously target metabolically distinct subpopulations and prevent niche-driven therapeutic escape.

**Table 6:** Metabolic heterogeneity across tumour compartments.

Feature	Cancer stem cells (CSCs)	Primary tumour bulk	Metastatic lesions	Therapeutic implication
Primary Fuel	OXPHOS/FAO	Glycolysis (core)/Mixed (periphery)	FAO (transit)/Glutaminolysis (niche)	Target OXPHOS in CSCs; address spatial heterogeneity in primary tumour; inhibit FAO at metastatic niche
Key Regulators	PGC-1α, STAT3, MYC (OXPHOS); lipid droplets (ferroptosis shield)	HIF-1α (hypoxic core); HIF-2α/MYC (periphery)	CD36 (FAO); GLS1 (glutaminolysis); organ niche metabolites	Biopsy from representative niches; spatial metabolomics for phenotype mapping
Metabolic Vulnerability	OXPHOS inhibitors (IACS-010759, metformin); GPX4 inhibitors	2-DG (glycolysis); AZD3965 (MCT1); HIF inhibitors	CPT1 inhibitors (etomoxir); CB-839 (GLS1) at metastatic sites	Sequential or combination regimens addressing distinct phenotypes; avoid monotherapy
Resistance Mechanism	Immune quiescence; lipid droplet ferroptosis shielding; high mitophagy flux	Metabolic switch to OXPHOS upon glycolysis inhibition; spatial biopsy bias	Organ niche metabolic symbiosis; primary tumour phenotype not predictive	Multi-region sampling; hyperpolarised 13C MRI for *in vivo* metabolic flux monitoring

#### 9.3.1 Tumour subtype-specific metabolic characteristics: a comparative analysis

Beyond the CSC–bulk–metastatic compartment framework, metabolic phenotypes vary substantially across histological tumour subtypes, generating subtype-specific therapeutic vulnerabilities. In acute myeloid leukaemia (AML), the metabolic landscape is fundamentally OXPHOS-dominant in leukaemic stem cells (LSCs), with electron transport chain Complex I providing the primary ATP source; LSCs are selectively eliminated by IACS-010759 while being resistant to glycolysis-targeted agents. In contrast, AML bulk blasts carrying IDH1/2 mutations operate via oncometabolite (2-HG)-driven epigenetic blockade rather than direct energy pathway dominance, making IDH inhibitors the rational first-line metabolic intervention. In diffuse large B-cell lymphoma (DLBCL), glycolytic dominance characterises the activated B-cell (ABC) subtype via constitutive NF-κB activation of GLUT1 and HK2, while germinal centre B-cell (GCB) DLBCL relies more on mitochondrial metabolism and is relatively AZD3965-resistant due to lower MCT1 dependency (31, 49).

In non-small cell lung cancer (NSCLC), metabolic phenotype is dictated by driver mutation: KRAS-mutant NSCLC upregulates glycolysis and macropinocytosis (amino acid scavenging), conferring sensitivity to macropinocytosis inhibitors and GLS1 blockade; LKB1 (STK11)-loss NSCLC — occurring in ~30% of KRAS-mutant cases — disrupts AMPK energy sensing, creating extreme OXPHOS dependency and sensitivity to phenformin (Complex I inhibitor) but resistance to mTOR inhibitors due to absent AMPK feedback. KEAP1/NRF2-mutant NSCLC (~20% of cases) exhibits PPP upregulation and GSH-driven multidrug resistance, requiring synthetic lethality approaches combining G6PD inhibition with ROS-inducing agents. In contrast, EGFR-mutant NSCLC relies on glycolytic-lipogenic co-activation via EGFR→PI3K/AKT/mTOR→SREBP1, and EGFR TKI resistance partially arises from metabolic rewiring toward FAO–OXPHOS, motivating combination with metformin or FASN inhibitors (28, 45).

In breast cancer, metabolic subtype divergence mirrors molecular classification: hormone receptor-positive (HR+) tumours activate ERα-driven glycolysis and FASN-mediated lipogenesis, with endocrine resistance frequently driven by metabolic reprogramming toward FAO and glutaminolysis upon estrogen deprivation. Triple-negative breast cancer (TNBC) exhibits the most pronounced Warburg phenotype, with high HK2 and LDH-A expression and glutamine addiction driven by MYC amplification, making GLS1 inhibition (CB-839) and LDH-A blockade rational combination partners. HER2-amplified breast cancer activates glycolysis via PI3K/AKT/mTOR but is distinguishable by enhanced lipid membrane biosynthesis to support receptor clustering, where FASN inhibition synergises with trastuzumab by disrupting HER2 lipid raft localisation. In hepatocellular carcinoma (HCC), Wnt/β-catenin-active tumours exhibit FAO upregulation (via WNT7B-driven CPT1 activation) in addition to glycolytic activation, creating a dual FAO+glycolysis phenotype distinct from the OXPHOS-dominant cholangiocarcinoma setting where IDH mutations are prevalent (8, 23, 35).

In renal cell carcinoma (RCC), VHL loss-driven pseudohypoxia creates constitutive HIF-2α stabilisation, locking tumour cells in a glycolytic-angiogenic programme specifically targetable by belzutifan. However, recent evidence from primary versus metastatic RCC demonstrates a critical subtype divergence: primary tumours show HIF-2α-dominant glycolytic phenotypes, while bone and lung metastases exhibit metabolic plasticity toward OXPHOS and glutaminolysis driven by the changed nutrient microenvironment at the metastatic niche — a finding with direct implications for the sequencing and combination of belzutifan with systemic metabolic inhibitors after metastatic progression. In IDH-mutant gliomas, the metabolic phenotype is uniquely shaped by 2-HG accumulation that drives CIMP-positive epigenetic silencing; metabolic imaging (hyperpolarised ^1^_3_C MRI) can directly detect 2-HG and monitor IDH inhibitor (vorasidenib) response *in situ*, representing the most advanced example of metabolism-guided precision oncology in clinical practice (9, 73).

## 10 Challenges, future directions, and conclusion

### 10.1 Challenges

Four fundamental challenges to the clinical translation of cancer metabolic therapeutics. (1) Metabolic plasticity and adaptive resistance. (2) Intra-tumoral metabolic heterogeneity. Metabolically diverse subpopulations are generated by clonal evolution and spatial gradients in oxygen and nutrients, and targeting a dominant phenotype allows the emergence of resistant metabolic clones. Cancer cells rapidly switch between glycolysis, OXPHOS, glutaminolysis, and FAO when a single pathway is targeted, with HIF-1α, MYC, and AMPK acting as metabolic state sensors. Third, patient stratification biomarkers: beyond IDH and VHL mutation status, reliable biomarkers stratifying patients by dominant metabolic wiring are lacking, leading to underpowered trials. Fourth, systemic toxicity: metabolic pathways essential in cancer are also critical in high-energy-demand normal tissues (cardiac, neural, hepatic), demanding tumour-selective delivery strategies (nanoparticles, prodrugs, TME-activatable agents) (14).

A fifth emerging challenge is the integration of systemic metabolism with tumour metabolic therapy. Obesity-associated hyperinsulinaemia chronically activates IGF-1R/insulin receptor → PI3K/AKT/mTOR, reducing the effective therapeutic window of mTOR inhibitors. Metformin, which activates AMPK via mitochondrial complex I inhibition, simultaneously targets tumour metabolism and improves insulin sensitivity, creating a dual systemic-tumour metabolic benefit particularly relevant in type 2 diabetes-associated cancers. Dietary interventions including caloric restriction mimetics (rapamycin, spermidine), ketogenic diets (reducing glucose availability for glycolytic tumours), and fasting-mimicking diets are being evaluated in clinical trials to enhance metabolic inhibitor efficacy (108–110).

A major biological limitation inherent to metabolic targeting is pathway redundancy and metabolic compensation. Unlike genetic oncogenic drivers (e.g., BCR-ABL, EGFR mutations) that represent single, non-redundant dependencies, cancer metabolism is characterised by interconnected, mutually compensatory pathways. Inhibiting glycolysis upregulates OXPHOS; blocking OXPHOS activates glycolysis and FAO; suppressing glutaminolysis induces pyruvate carboxylation and alternative anaplerotic routes. This inherent redundancy means that monotherapy metabolic interventions are unlikely to achieve durable responses in the majority of patients, and rational combination strategies—while scientifically compelling—carry compounded toxicity risks that limit clinical dose escalation (27, 28).

A critical translational limitation is the absence of validated predictive biomarkers for most metabolic inhibitors beyond IDH mutation status and VHL loss. Patient selection for metabolic trials currently relies on genomic proxies (e.g., KRAS mutation for macropinocytosis dependency; LKB1 loss for AMPK-mTOR vulnerability) rather than direct metabolic flux measurements, leading to clinically heterogeneous trial populations and diluted effect sizes. Single-cell and spatial metabolomics technologies that could provide direct metabolic stratification remain in early translational stages and are not yet suitable for prospective clinical deployment. Furthermore, tumour metabolic phenotype can evolve substantially between biopsy time points, primary and metastatic sites, and under therapeutic pressure, making static biomarker assessment potentially misleading (8, 85).

The clinical translation of metabolic inhibitors has also revealed tumour-type and context-specific limitations. FAO inhibitors that show preclinical efficacy in AML and breast cancer models have demonstrated narrow therapeutic windows due to cardiac mitochondrial FAO dependence. OXPHOS inhibitors (IACS-010759) caused dose-limiting peripheral neuropathy in Phase I trials. GLS1 inhibitor monotherapy (CB-839) showed modest single-agent activity despite strong preclinical rationale, underscoring that metabolic dependencies observed in cell lines and mouse models do not reliably translate to patient tumours growing in complex physiological microenvironments. Resolving these translational gaps will require patient-derived organoid metabolic profiling, improved preclinical models incorporating stromal and immune complexity, and adaptive clinical trial designs that incorporate metabolic response monitoring via hyperpolarised ¹³C MRI or PET metabolic imaging (49, 50).

### 10.2 Future directions and conclusion

The central thesis of this review is that metabolic plasticity, the dynamic, context-dependent ability of cancer cells to reprogram their metabolic state in response to oncogenic signals, microenvironmental pressures, and therapeutic challenge, is the fundamental driver of tumour progression, immune evasion, and therapeutic resistance. Metabolic reprogramming is a fundamental, multidimensional hallmark of cancer orchestrated by an intricate network of molecular mediators spanning oncogenic transcription factors, tumour suppressors, signalling kinases, metabolic enzymes, mitochondrial dynamics, and immune crosstalk. Clinical translation has been demonstrated by IDH inhibitors, belzutifan, KRAS inhibitors, and mTOR rapalogs. The intersection of metabolic reprogramming with epigenetic regulation via metabolite cofactors (acetyl-CoA, SAM, α-KG, 2-HG, lactyl-CoA) creates additional drug nodes where epigenetic and metabolic inhibition can be synergistically combined. Future advances require: (i) AI-GSMM-driven metabolic patient stratification; (ii) single-cell and spatial metabolomics-guided trial design identifying metabolic niche-specific vulnerabilities; (iii) rational combination strategies targeting multiple metabolic nodes to preempt adaptive escape; (iv) integration of circadian pharmacology, host metabolic comorbidity management, and TME metabolic normalisation; and (v) next-generation biomarker development for predictive patient enrichment. A global understanding of the molecular mediators of metabolic reprogramming offers a transformative opportunity to selectively eradicate cancer cells while sparing normal tissue metabolism to further the precision oncology agenda.

## Glossary

2-HG: 2-hydroxyglutarate
ACLY: ATP-citrate lyase
AhR: aryl hydrocarbon receptor
AML: acute myeloid leukemia
AMPK: AMP-activated protein kinase
BMAL1: brain and muscle ARNT-like protein 1
CAF: cancer-associated fibroblast
CAR-T: chimeric antigen receptor T cell
CIMP: CpG island methylator phenotype
CSC: cancer stem cell
DC: dendritic cell
DNL: *de novo* lipogenesis
DRP1: dynamin-related protein 1
ETC: electron transport chain
FAO: fatty acid oxidation
FASN: fatty acid synthase
G6PD: glucose-6-phosphate dehydrogenase
GLS1: glutaminase 1
GPX4: glutathione peroxidase 4
GSMM: genome-scale metabolic model
HIF: hypoxia-inducible factor
HK2: hexokinase 2
HDAC: histone deacetylase
HAT: histone acetyltransferase
IDH: isocitrate dehydrogenase
ICI: immune checkpoint inhibitor
IDO1: indoleamine 2,3-dioxygenase 1
KDM: lysine demethylase
LDH-A: lactate dehydrogenase A
MCT: monocarboxylate transporter
mtDNA: mitochondrial DNA
MYC: MYC proto-oncogene
NAD+: nicotinamide adenine dinucleotide
NK: natural killer (cell)
NRF2: nuclear factor erythroid 2-related factor 2
OXPHOS: oxidative phosphorylation
PGC-1α: peroxisome proliferator-activated receptor-gamma coactivator 1-alpha
PKM2: pyruvate kinase M2
PPP: pentose phosphate pathway
PPAR: peroxisome proliferator-activated receptor
RCC: renal cell carcinoma
SAM: S-adenosylmethionine
SCENITH: single cell energetic metabolism by profiling translation inhibition
SREBP: sterol regulatory element-binding protein
TAM: tumour-associated macrophage
TCA: tricarboxylic acid
TET: ten-eleven translocation
TME: tumour microenvironment
VEGF: vascular endothelial growth factor
WT: wild-type
YAP: yes-associated protein.
